# CXCR4-dependent macrophage-to-fibroblast signaling contributes to cardiac diastolic dysfunction in heart failure with preserved ejection fraction

**DOI:** 10.7150/ijbs.65802

**Published:** 2022-01-09

**Authors:** Ning Zhang, Qunchao Ma, Yayu You, Xiangyang Xia, Cuiping Xie, Yuxue Huang, Zhuo Wang, Feiming Ye, Zhaosheng Yu, Xiaojie Xie

**Affiliations:** 1 Department of Cardiology, Cardiovascular Key Laboratory of Zhejiang Province, Second Affiliated Hospital, Zhejiang University College of Medicine, 88 Jiefang Rd, Hangzhou, Zhejiang Province, 310009, PR China; 2 State Key Laboratory of Fluid Power and Mechatronic Systems, Department of Mechanics, Zhejiang University, Hangzhou 310027, China

**Keywords:** CXCR4, macrophages, HFpEF, inflammation, fibrosis

## Abstract

**Rationale:** Heart failure with preserved ejection fraction (HFpEF) can arise from hypertension‐induced cardiac remodeling. Monocyte/macrophage accumulation and inflammation are crucial elements in the pathogenesis of hypertension-induced cardiac remodeling. The C-X-C chemokine receptor 4 (CXCR4) is a critical regulator of the macrophage-mediated immune response. Nevertheless, the contribution of CXCR4 to macrophage phenotype and function during the progression of HFpEF remains unclear. Herein, we aimed to determine the role of macrophagic CXCR4 in heart failure with preserved ejection fraction (HFpEF).

**Methods:** As a HFpEF model, wild type mice and myeloid-specific CXCR4 deficiency mice were subjected to pressure overload for 30 days to assess the function of macrophagic CXCR4 on cardiac function. Medium from macrophages was used to treat cardiac fibroblasts to study macrophage-to-fibroblast signaling.

**Results:** We found circulatory CXCR4+ immune cells, mainly monocytes, markedly increased in HFpEF patients with hypertension. In the experimental HFpEF mice model, macrophages but not neutrophils represent the main infiltrating inflammatory cells in the heart, abundantly expressing CXCR4. Myeloid-specific CXCR4 deficient impeded macrophage infiltration and inflammatory response in the heart of HFpEF mice, thus ameliorating cardiac fibrosis and improving cardiac diastolic function. Furthermore, transcriptomic profiling data revealed that CXCR4 loss in macrophages exhibited a decreased transcriptional signature associated with the regulation of inflammatory response. Notably, CXCR4 significantly augmented chemokine (C‑X‑C) motif ligand (CXCL3) expression, which at least partly contributed to fibrosis by promoting myofibroblast differentiation. Mechanistically, the increased production of pro-inflammatory cytokines in CXCR4 expressed macrophages could be attributed to the suppression of the peroxisome proliferator-activated receptor γ (PPARγ) activity.

**Conclusions:** Collectively, our data supported that the infiltration of CXCR4+ macrophages in the heart exacerbates hypertension-induced diastolic function by promoting pro-inflammatory cytokines production and thus may serve as a potential therapeutic target for hypertension-induced HFpEF.

## Introduction

Heart failure with preserved ejection fraction (HFpEF), characterized by cardiac diastolic dysfunction, accounts for approximately half of the overall heart failure cases. However, the precise etiopathogenesis underlying HFpEF is still unknown. Patients with HFpEF do unfortunately not benefit from the classic medication regimens of heart failure with reduced ejection fraction (HFrEF) and always have a poor clinical prognosis. Thus, it is of great importance to unravel the mechanisms and seek therapeutic targets of HFpEF.

HFpEF is associated with multiple risk factors, such as advancing age, hypertension and diabetes 1, 2. Up to 90% of HFpEF patients suffer from hypertension 3-5. Recent studies have shed light on the inflammation and fibrosis in HFpEF 6-8. On the one hand, the increase of cardiac workload during HFpEF promotes the infiltration of inflammatory cells into the heart 9. On the other hand, the excessive workload stimulates inflammatory cytokines and chemokines in cardiac tissue, leading to extracellular matrix deposition and diastolic dysfunction 10. Macrophage-mediated inflammation plays a pivotal role throughout the entire course of heart failure. It chiefly contributes to cardiac injury in the initial stage and fibrosis in the late stage 10-12. Therefore, macrophage-oriented intervention could be a promising candidate for the novel therapeutic strategy of HFpEF.

Emerging evidence indicates monocytes/macrophages trafficking in damaged tissue during hypertension, predominately controlled by C-X-C motif chemokine ligands (MCP-1, IL-8, CCL5, and CX3CL1) and their receptors (CCR2, CXCR1, CCR5, and CX3CR1)^13^-^15^. CXCR4, known as a chemokine receptor, mediates inflammatory cells infiltration to the heart after myocardial infarction 16, 17. Current research has indicated that CXCR4 antagonist AMD3100 facilitates angiogenesis and improves cardiac contractile function after myocardial infarction 18. Besides, CXCR4 blocker POL5551/6326 improved cardiac function in mice and pigs after I/R by reducing mobilization of splenic regulatory T cell 19. Of note, these conclusions are based on systemic suppression of CXCR4, making it an attractive target for therapeutic intervention. Nevertheless, the primary source of CXCR4 and its mechanism that contributes to HFpEF has not been fully elucidated.

The limited availability of mouse models has impeded the exploration of potential treatments for HFpEF 20,10. Previous studies have shown that SAUNA (All mice underwent uninephrectomy and received either a continuous infusion of saline (Sham) or d‐aldosterone (0.30μg/h) via osmotic minipumps and salty (1% NaCl) drinking water for 30 days.) is a suitable animal model for HFpEF, albeit it only represents some hypertensive patients 10, 21. This study aimed to define the role of macrophagic CXCR4 in the pathogenesis of HFpEF. To this end, we subjected wild-type (WT) mice and myeloid-specific CXCR4-deficient (MKO) mice to SAUNA to establish the mice HFpEF model. As a result, we firstly uncover the potent effects of macrophagic CXCR4 in inflammation and cardiac fibrosis in SAUNA-induced HFpEF mice model, which was achieved via promoting macrophage infiltration and pro-inflammatory cytokine production.

## Materials and Methods

### Human samples

This investigation was performed according to the Declaration of Helsinki and with approval by the Institutional Ethics Committee of Second Affiliated Hospital of Zhejiang University. Twenty-three patients with HFpEF and thirteen normal individuals were included in the study. Human blood samples were collected from patients with HFpEF and normal individuals enrolled at Second Affiliated Hospital. Blood was erythrocyte-depleted with RBC lysis buffer (R1010, Solarbio, Beijing, China) and incubated at room temperature in PBS with antibodies against human CD45(557659, BD Biosciences, San Jose, CA, USA), CD11b(550019, BD Biosciences, San Jose, CA, USA), CD66B(305118, BioLegend, San Diego, CA, USA), CXCR4(551510, BD Biosciences, San Jose, CA, USA). Flow cytometry assay was performed using a BD FACS Calibur FACS machine (BD Biosciences, San Jose, CA, USA).

### Mice

All animal procedures were approved by the Animal Policy and Welfare Committee of Second Affiliated Hospital, College of Medicine, Zhejiang University.

LysM Cre^+/-^ mice and CXCR4^Flox/Flox^ mice were purchased from Jackson Lab (Bar Harbor, ME, USA). In LysM Cre^+/-^ mice, the Cre protein is regulated by the myeloid cell-specific promoter LysM. LysM Cre^+/-^ mice were crossbred with CXCR4^Flox/Flox^ mice to generate CXCR4^Flox/Flox^/LysM Cre^+/-^ mice (MKO) which myeloid cells were specific knockout CXCR4 gene.

Wild-type (WT) mice and MKO mice were bred in-house under specific pathogen-free conditions with free access to a regular chow diet and water, at a constant temperature (22±2°C) and humidity (60%-65%) with a 12h dark/light cycle. All mice were maintained on C57BL/6 background and 8 weeks old male WT mice and MKO mice were conducted to do further studies at randomization.

### Identification of transgenic mice

Genomic DNA of mouse tail was extracted by phenol-chloroform method and used as the template for polymerase chain reaction (PCR) genotyping. Primers used for genotyping are listed in Table S1.

### *In vivo* interventions

Mouse model of SAUNA was performed on 6-8 weeks old male C57BL/6 mice according to the previous study 11. All mice underwent uninephrectomy and received either a continuous infusion of saline or d‐aldosterone (0.3ug/h, Sigma‐Aldrich Co., St. Louis, Missouri, 706035) via osmotic minipumps (Alzet, Durect Corp, Cupertino, CA) and salty (1% NaCl) drinking water for 30 days.

### Bone marrow transplantation

10-week-old C57BL/6 male mice were fed with medicated water supplemented with 200mg/m sulfamethoxazole and 40mg/ml trimethoprim oral suspension before irradiation for one week. The animals received 8.5Gy total body irradiation for at least 6h before injecting the bone marrow cells. 10^7^cells/mice were then injected intravenously to rescue the hematopoietic system of the irradiated mice. The mice were then exposed to SAUNA four weeks after the transplantation.

### Echocardiography Analysis of Cardiac Function

The mice were subjected to transthoracic two-dimensional M-mode echocardiography to evaluate cardiac function using a Vevo 2100 system (Visual Sonics, Canada) equipped with an 80-MHz probe as described previously 22. Mice were properly positioned and stabilized on a bench at 37°C, followed by anaesthetization using isoflurane. The long axis and short axis 2- dimensional (2D) B-mode tracing was then applied. The probe was positioned at a mid-papillary muscle level, followed by recording the M-mode tracing images. The obtained images were then used to measure the wall thickness of the left ventricle and the diameter of the chambers. We measured and analyzed three consecutive cardiac cycles that were not affected by respiration and recorded the early peak diastolic blood flow (E wave) and late diastolic blood flow (A wave) to evaluate the diastolic function. The tissue doppler module was used to evaluate the myocardial motion spectrum of the ventricular septal mitral annulus, which helped measure the early diastolic exercise rate (E' wave) and late diastolic exercise rate (A'wave). In addition, the percentage of fraction shortening (FS%) was calculated to assess systolic function.

### Exercise Capacity

Mice were run according to previous studies 23, 24. We assessed the ability of untrained mice to run for distance. The rod coordinator of Med Associates, Inc. was used to measure the motor coordination ability of mice. The mice were first placed on a stationary rotating stick instrument facing the wall for two minutes for adaptation. The instrument's rotating speed was then set to 0-30 cm/min, with a uniform acceleration time of the 90s and a rotating time of five minutes. In this experiment, a mouse will run continuously or fall when the stick starts rotating. The stick stops automatically once the mouse is dropped. Therefore, the system can record the time that the mouse stays mobile on the stick. The discontinuity measurement method was used to measure when the mouse is mobile three times at an interval of two h, with the average of the three measurements being recorded.

### Hemodynamic measurements

According to described previously 21, the closed-chest approach was used to obtain the LV hemodynamics in anesthetized mice using a Millar MPVS-300 system equipped with a Millar SPR-839 catheter. At the end of the experiment, the anesthetized mice were fixed on the operating table, and their neck hair was cut. The ventral side of the neck was cut longitudinally to the right, and the right common carotid artery was isolated on the inside of the sternocleidomastoid muscle. A wedge-shaped incision was then cut, followed by inserting the catheter into the LV through the right carotid artery. The following indicators were used for the continuous monitoring of hemodynamics. Left ventricular end-systolic pressure (LVSP) and left ventricular end-diastolic pressure (LVEDP), and left ventricular systolic and diastolic rate (±dp/dt). The collected data were then analyzed using the Lab Chart Pro software (AD Instruments).

### Heart quality index

On the 30th day after the operation, the mice were weighed, the mice were anesthetized with chloral hydrate, the chest was opened, and the heart was taken out. The heart was placed in PBS to remove the residual blood and adipose tissue, and the heart tissue was blotted with absorbent paper and weighed. Calculate the ratio of the heart (mg) to body weight (g).

### Flow cytometry

The hearts were extensively flushed using PBS and then excised. The remote myocardium was then separated using a dissection microscope followed by mincing using a pair of scissors and digestion using collagenase IV (2mg/ml) (Sigma-Aldrich) at a speed of 100rpm for 1 h at 37°C. The hearts were subsequently homogenized through a 40-μm nylon mesh. For immune cells staining, cell suspensions were labeled using biotin-conjugated anti-mouse antibodies directed against CD45(555482, BD Biosciences, San Jose, CA, USA), CD11b (557657, BD Biosciences, San Jose, CA, USA),CCR2(150608, BD Biosciences, San Jose, CA, USA),Ly6G(560602, BD Biosciences, San Jose, CA, USA), F4/80(123108, 123114, BD Biosciences, San Jose, CA, USA), and CXCR4 (551966, BD Biosciences, San Jose, CA, USA).

### Immunofluorescent staining

Mice were anesthetized, and the hearts were removed, weighed, and cut into transverse slices through the middle of the ventricles between the atrioventricular groove and the apex. Heart tissues obtained on the 30th-day post SAUNA were dehydrated in 30% sucrose solution (prepared in PBS) and embedded in Tissue-Tek OCT (Sakura Finetek USA Inc., Torrance, CA, USA) compound and snap-frozen in liquid nitrogen. For immunohistochemistry, frozen tissue sections (7.0 μm thick) were fixed in 4% paraformaldehyde, permeabilized in 0.2% Triton X-100 for 10 min each, blocked with PBS containing 3% bovine serum albumin (BSA). The slides were incubated with antibodies against F4/80 (6640, Abcam, Cambridge, MA, USA), CXCR4 (181020, Abcam, Cambridge, MA, USA), aSMA(Cell Signaling Technology, Danvers, MA, USA), CXCR2 (14935, Abcam, Cambridge, MA, USA) and CXCL3 (220431, Abcam, Cambridge, MA, USA), overnight at 4 °C, followed with incubation of secondary antibody for 1 h at room temperature. Finally, the heart sections were mounted with DAPI-containing anti-fade medium and imaged under fluorescence microscopy.

### Masson staining

Freshly isolated hearts were fixed in 4% paraformaldehyde and sectioned into sections of 7 μm. The paraffin sections are placed in an oven at 60 ° C for 1 to 2 h. The Masson staining was performed using Masson's Trichrome Stain Kit (Solarboi LIFE SCIENCE, USA). The heart paraffin section was fixed in 4% paraformaldehyde and dewaxed to water. Then, a hematoxylin dyeing solution was used for 5 to 10 minutes. The water was slightly washed, and 1% hydrochloric acid was differentiated. Rinse in running water for a few minutes. Masson complex staining solution stained for 5 to 10 minutes. Rinse the distilled water slightly. 1% phosphotungstic acid solution was treated for about 5 minutes. There is no need to wash directly dyed with bright green staining solution (or blue aniline solution) for 5 min.1% glacial acetic acid water treatment for 1 min. 95% alcohol dehydration multiple times. Dehydration of anhydrous ethanol, transparent xylene, and sealed by neutral gum. The results are shown below. Collagen fibers are green (stained with bright green) or blue (stained with aniline blue), cytoplasm, muscle fibers, red blood cells are red, and the nucleus is blue.

### Cell culture

Bone marrow-derived macrophages were obtained and cultured from MKO and WT mice. Cells were stimulated during their differentiation to macrophages with 10ng/ml Recombinant mouse M-CSF (315-02, PEROTECH, New Jersey, USA) 7 days after isolation. Cells were stimulated with 1ug/ml HMGB1 (ab181949, Abcam, Cambridge, MA, USA) and 100ng/ml CXCL12 (ab270067, Abcam, Cambridge, MA, USA) for 12 h. Cells were harvested for protein extraction or RNA isolation with an RNA isolation kit.

### RNA sequencing

Total RNA was extracted according to the instruction manual of TRIzol® 1 (Life Technologies, Inc., Gaithersburg, MD) from whole heart tissue of WT mice subjected to sham and SAUNA for 30 days. Preparation of library and sequencing of the transcriptome was performed by illumina novaseq 6000 (Novogene Bioinformatics Technology Co., Ltd., Beijing, China).

Total RNA was extracted according to the instruction manual of TRIzol® 1 (Life Technologies, Inc., Gaithersburg, MD) from macrophages derived from MKO and WT mice stimulated with 1ug/ml HMGB1 (BioLegend, San Diego, CA, USA) for 12 h. Preparation of library and sequencing of the transcriptome was performed by illumina novaseq 6000 (Novogene Bioinformatics Technology Co., Ltd., Beijing, China).

### Western blot

Western blotting was performed to quantify specific protein expression levels in heart tissue, macrophages, and primary cardiac fibroblast (CF). Samples were lysed with RIPA buffer containing protease inhibitor cocktail (Beyotime, China). The protein concentration was determined by BCA assay (23225, Pierce, Thermo Fischer, IL, USA). Equal quantities of protein were loaded and run on SDS-PAGE gels and then transferred to polyvinylidene difluoride (PVDF) membranes. Each membrane was blocked in 5% BSA and subsequently incubated overnight at 4°C with primary antibodies including NF-κB p65 (8242, Cell Signaling Technology, Danvers, MA, USA), pNF-κB p65 (3033, Cell Signaling Technology, Danvers, MA, USA), MEK (ET1602-3, Huabio, China), pMEK (ET1612-40, Huabio, China), Erk (RT1484, Huabio, China), pErk (ET1603-22, Huabio, China), aSMA (48938, Cell Signaling Technology, Danvers, MA, USA), Fibronectin (2413, Abcam, Cambridge, MA, USA), PPARγ (2435, Cell Signaling Technology, Danvers, MA, USA), and CXCR2 (14935, Abcam, Cambridge, MA, USA). After washing, the membranes were incubated with horseradish peroxidase-conjugated goat anti-mouse or anti-rabbit secondary antibody (Invitrogen, USA) for 1h at room temperature and detected with an enhanced chemiluminescent kit (Millipore). Glyceraldehyde-3-phosphate dehydrogenase (GAPDH) (5174, Cell Signaling Technology, Danvers, MA, USA) was used as a reference. Image analysis and blot quantification were performed with Image Quant LAS 4000 mini biomolecular imager (GE Healthcare, Uppsala, Sweden).

### Real-time PCR

Total RNA was isolated from the heart, CFs, and macrophages by Trizol reagent (Invitrogen, Carlsbad, CA, USA) per the manufacturer's protocols. The cDNA was synthesized from 1 μg of RNA with Moloney Murine Leukemia Virus reverse transcriptase and oligo (dT) 18 primer. According to the manufacturer's instructions, a quantitative RT-PCR (Q-PCR) was performed using the SYBR PCR master mix in the ABI Step One-Plus Detection system (Applied Biosystems, USA). PCR conditions were 95°C for 10 min and 40 cycles of 95°C for 30 s, 60°C for 30 s and 72°C for 1 min. 18s (for mRNA) was served as a control, and the target gene expression was calculated by the 2^-ΔΔCT^ method comparative method. The primers were listed in Table S1.

### Enzyme-linked immunosorbent assay (ELISA)

The supernatants of heart tissue lysates and the conditioned medium of macrophages were collected to detect CXCL3 ( CSB-EL00624MO, CUSABIO, Wuhan, Hubei, China) and CXCL12 (MCX120, R&D Systems, Minneapolis, MN, USA) according to the enzyme-linked Immunosorbent Assay Kit protocol.

### Isolation of neonatal mice primary cardiac fibroblasts

Neonatal mice were immersed in a 75% ethanol tank for 5 seconds and then transferred to an ultra-clean platform. They were fixed to a sterile foam board with tacks and disinfected with povidone-iodine. Cut the skin, and expose the chest slightly with eye iris scissors on the left side of the midline of the xiphoid. Use the scissors to press the right edge of the sternum to make the heart jump out naturally. Hook the root of the heart with curved forceps, remove the heart, and put it into a petri dish containing pre-chilled PBS solution. After the hearts of all neonatal mice were removed, connective tissue, fat, and blood vessels were removed from the heart, and bloodstains were removed by pre-chilling the PBS solution 3 times. The heart was cut into tissue pieces of about 1mm × 1mm × 1mm, added to a 50ml sterile centrifuge tube filled with type II (2mg/ml, Sigma-Aldrich)digestion fluid, digested in a constant temperature shaker at 37 ° C for 10 minutes at 100 rpm. The supernatant was collected and used Neutralize with 10% FBS in the low-sugar medium. Add 10ml digestive enzyme solution and digest for 10 minutes. Repeat the above steps 5 times. The cell suspensions collected at different stages were uniformly pipetted, centrifuged at 1200 rpm/min for 5 minutes, the supernatant was discarded, and the cell pellet was resuspended in a low-sugar medium containing 10% FBS. They were placed in 5% CO2, 37°C incubators for 1.5h. Discard the medium and add a new low-glucose medium containing 10% FBS to adherent cells (mainly cardiac fibroblasts) for further culture.

### Primary cardiac fibroblasts treatment

The conditioned medium was collected from MΦ^WT^ or MΦ^MKO^ treated with either HMGB1 or vehicle. CFs were seeded in 12-well plate, added the conditioned medium, and cultured for 24 h. As specified, anti-CXCL3 neutralizing antibody (0.5μg, AF5568, R&D, Minneapolis, MN, USA) or recombinant Mouse CXCL3 (10ng/ml, HY-P7153, MCE, Shanghai, China) was added into the medium, with or without CXCR2 inhibitor SB225002 (10nM, HY-16711, MCE, Shanghai, China) pre-treatment for 1h. The medium was replaced with fresh serum-free DMEM. After cultured for another 24h, cells were harvested for protein extraction or RNA isolation with an RNA isolation kit.

### Statistical analysis

Results are expressed as mean ± SEM. All experiments were conducted in at least three biological replicates unless otherwise indicated. N numbers indicate biological replicates of experiments performed at least three times unless otherwise indicated. Comparison between two groups was calculated using unpaired, two-tailed student's t-test. For comparison of multiple experimental groups, either one-way ANOVA or two-way ANOVA was performed where appropriate. Dunnett's multiple comparisons post-test or Bonferroni's post-test was performed where applicable after performing multiple comparisons with ANOVA. A significant difference was achieved when the overall p-value was < 0.05.

## Results

### Macrophages highly expressing CXCR4 accumulate in SAUNA-induced heart

The HFpEF model was successfully established by using the SAUNA protocol^10^, which was reflected by a lower early to late mitral inflow velocity ratio (E/A), a higher transmitral to mitral annular early diastolic velocity ratio (E/E') (Figure S1A), an increased left ventricular filling pressure (Figure S1B), a significant reduction in running time (Figure S1C), an enlargement of cardiac hypertrophy (Figure S1D and 1E), and an elevation of cardiac fibrosis (Figure S1F ).

**Figure 1:**
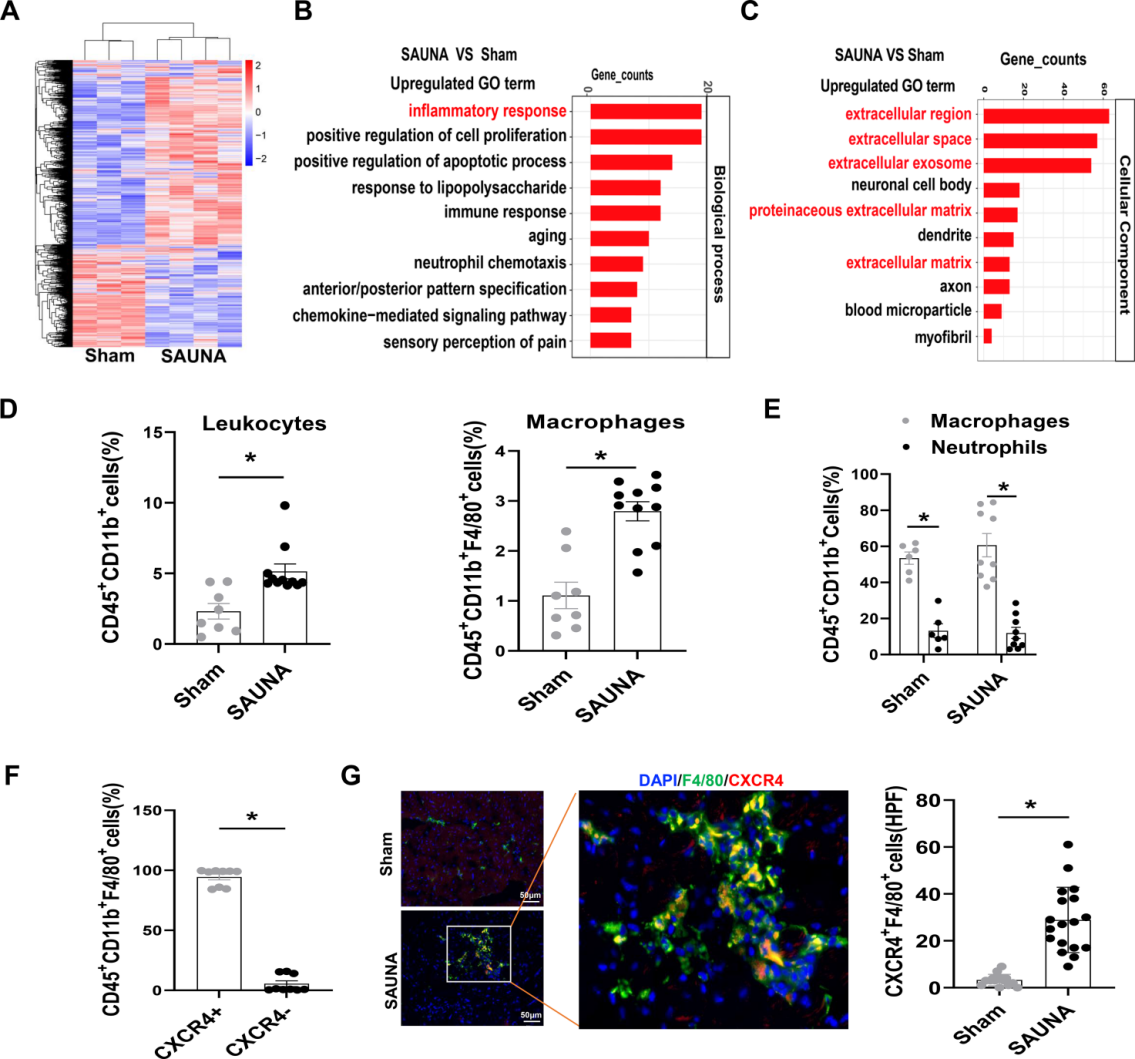
** Macrophages highly expressing CXCR4 accumulate in SAUNA-induced heart and circulatory CXCR4^+^ cells are increased in patients with HFpEF. (A)** Heartmap for differentially expressed genes, as identified by transcriptomic profiling, in SAUNA hearts compared with sham hearts. Sham, n=3; SAUNA, n=4. **(B)** GO enrichment analysis for biological process in SAUNA hearts compared with sham hearts. Top 10 of enriched GO term are listed. Sham, n=3; SAUNA, n=4. **(C)** GO enrichment analysis for cellular component in SAUNA hearts compared with sham hearts. Top 10 of enriched GO term are listed. Sham, n=3; SAUNA, n=4. **(D)** Flow cytometry analysis of CD45+ CD11b+leukocytes, and CD45+CD11b+ F4/80+ macrophages in the heart of sham and SAUNA group. Sham, n=8; SAUNA, n=11. **(E)** Flow cytometry analysis the percentage of macrophages and neutrophils in CD45+CD11b+cell. Sham, n = 6; SAUNA, n = 8. **(F)** Flow cytometry analysis of CXCR4+ population in CD45+CD11b+ F4/80+ cells. **(G)** Immunofluorescence staining of anti-CXCR4 (red) and anti-F4/80 antibody (green) (DAPI, blue) in the heart of sham and SAUNA group, respectively, Scale bar =100μm. Sham, n=6; SAUNA, n=8. GO, Gene Ontology; All data were analyzed using unpaired two-tailed student's t-test. * p<0.05.

To broadly explore the pathogenesis of SAUNA-induced HFpEF, we first examined genome-wide transcriptional changes in the heart of SAUNA mice by performing microarray analysis. The heart of SAUNA mice exhibited significant differences in gene abundances compared to sham subjects (Figure 1A). Gene ontology (GO) analysis revealed that the markedly upregulated genes were focused on the inflammatory response (Figure 1B), and primarily enriched in the extracellular region (Figure 1C). Further, we discovered that the number of immune cells (Figure S2) such as leukocytes and macrophages was significantly increased in SAUNA-exposed heart (Figure 1D). Those infiltrating cells consisted predominantly of macrophages and few neutrophils (Figure 1E). This was paralleled with the elevation of CCR2+ bone marrow-derived macrophages in SAUNA-exposed heart (Figure S3). Notably, we observed that a chemokine receptor, CXCR4, was highly expressed in the infiltrated macrophage (Figure 1F), which was further supported by immunofluorescence staining (Figure 1G). Hence, these data elucidated that macrophages that were highly expressing CXCR4 accumulate in the SAUNA exposed-heart during the progression of HFpEF.

### Myeloid-specific CXCR4-deficient attenuates SAUNA-induced cardiac diastolic dysfunction, hypertrophy, and fibrosis

Myeloid-specific CXCR4-deficient (MKO) mice were generated, and CXCR4 was markedly knockdown in macrophages from MKO mice compared with wild-type (WT) mice (Figure S4). Besides, the blood routine exhibited that the numbers of circulatory leukocytes, including lymphocytes, neutrophils, monocytes, eosinophils, and eosinophils of MKO mice were similar to WT mice at baseline (Figure S5).

**Figure 2:**
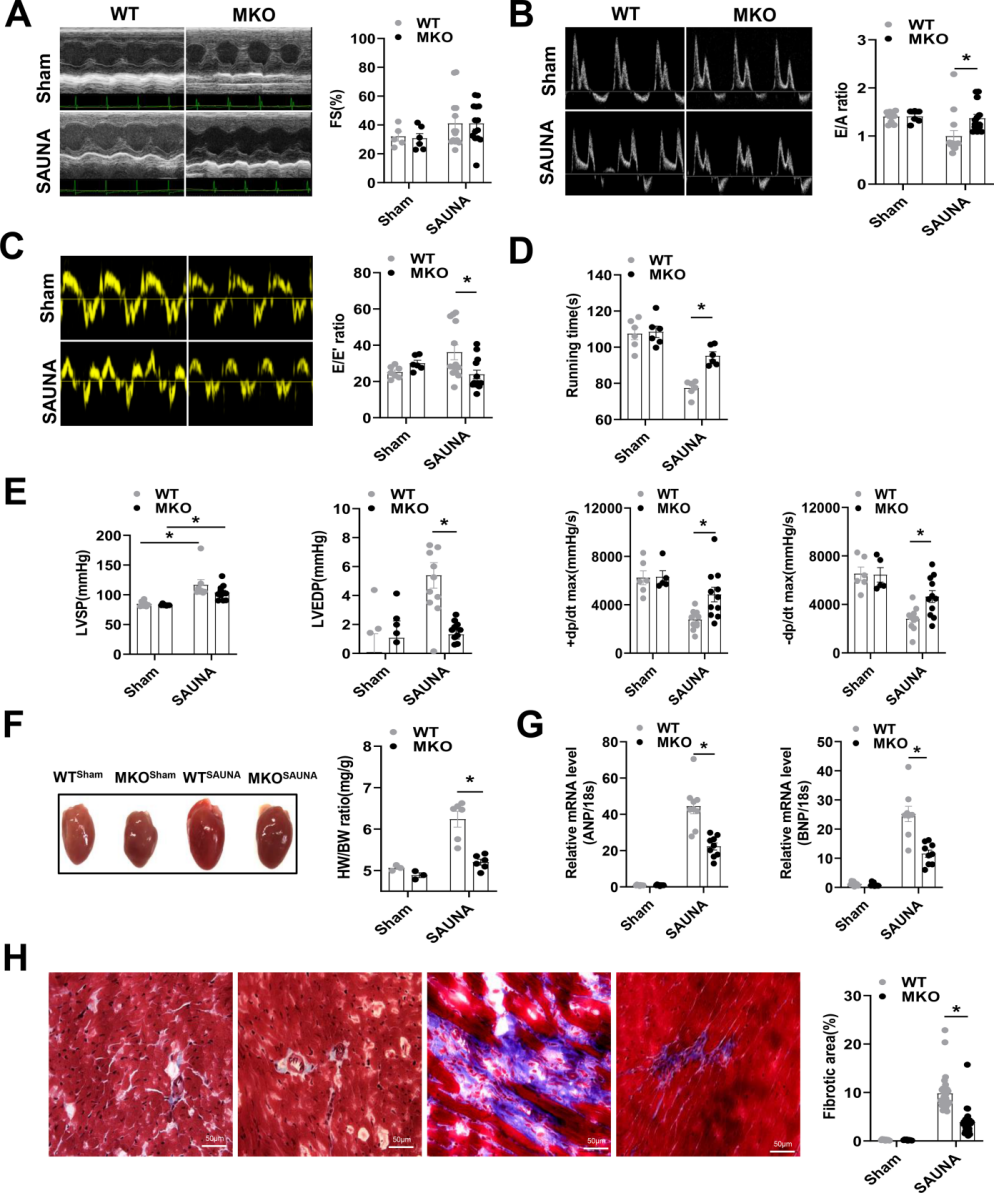
** Absence of CXCR4 in macrophages protects against SAUNA-induced cardiac hypertrophy, fibrosis, and diastolic dysfunction. (A-C).** Representative echocardiographic and measurement of FS%, E/A, and E/E' of WT mice and MKO mice after sham or SAUNA operation. WT^sham^, n=6; MKO^sham^, n=6; WT^SAUNA^, n=14; MKO^SAUNA^, n=14. **(D)** Recording of running times during the exercise exhaustion test of WT and MKO mice after sham or SAUNA operation. WT^sham^, n=6; MKO^sham^, n=6; WT^SAUNA^, n=6; and MKO^SAUNA^, n=6. **(E)** The measurement of LVEDP, LVSP, +dp/dt, and -dp/dt in WT and MKO mice after sham or SAUNA operation. WT^sham^, n=6; MKO^sham^, n=6; WT^SAUNA^, n=14; and MKO^SAUNA^, n=14. **(F)** Representative heart size and HW/BW in WT and MKO mice after sham or SAUNA operation. WT^sham^, n=3; MKO^sham^, n=3; WT^SAUNA^, n=6; and MKO^SAUNA^, n=6. **(G)** QPCR analysis of ANP and BNP in the heart of WT and MKO mice after sham or SAUNA operation. WT^sham^, n=9; MKO^sham^, n=9; WT^SAUNA^, n=9; and MKO^SAUNA^, n=9. **(H)** Masson's trichrome staining of heart tissues and quantification of the fibrotic area in WT and MKO mice after sham or SAUNA operation. Scale bars=50µm. WT^sham^, n=6; MKO^sham^, n=6; WT^SAUNA^, n=6; and MKO^SAUNA^, n=6. WT, wild-type; MKO, myeloid-specific CXCR4-deficient; LVEDP, left ventricular end-diastolic pressure; LVSP, left ventricular systolic pressure; +dp/dt, maximal rate of increase of left ventricular pressure; -dp/dt, maximal rate of the decrease of left ventricular pressure; HW/BW, the ratio of heart weight to body weight; ANP, atrial natriuretic peptide; BNP, brain natriuretic peptide. All data were analyzed using two-way ANOVA with Bonferroni's multiple comparisons test.*. p<0.05.

Then, WT and MKO mice were administered SAUNA to explore the role of macrophagic CXCR4 in HFpEF. Echocardiography was performed on day 30 after SAUNA. Cardiac contractile function reflected by left ventricular fraction shortening (FS%) was not affected by SAUNA in both the WT and MKO mice (Figure 2A). However, MKO mice exposed to SAUNA exhibited a noticeable improvement of diastolic function reflected by the higher E/A ratio (Figure 2B), lower E/E' ratio (Figure 2C), and increased tolerance to exercise (Figure 2D) compared to WT mice. Moreover, MKO mice displayed lower left ventricular end-diastolic pressure (LVEDP), higher maximal rate of the increase of left ventricular pressure (+dp/dt), and maximal rate of the decrease of left ventricular pressure (-dp/dt) (Figure 2E) compared to WT mice. The deficiency of CXCR4 in macrophages also alleviated cardiac remodeling, reflected by narrowed heart size, decreased HW/BW ratio (Figure 2F) as well as reduced ANP and BNP expression compared to WT mice (Figure 2G). Furthermore, fibrosis was profoundly inhibited in MKO mice with myeloid-specific CXCR4 deficiency (Figure 2H). These results provide evidence supporting the cardioprotective role of myeloid-specific CXCR4 deficiency.

**Figure 3:**
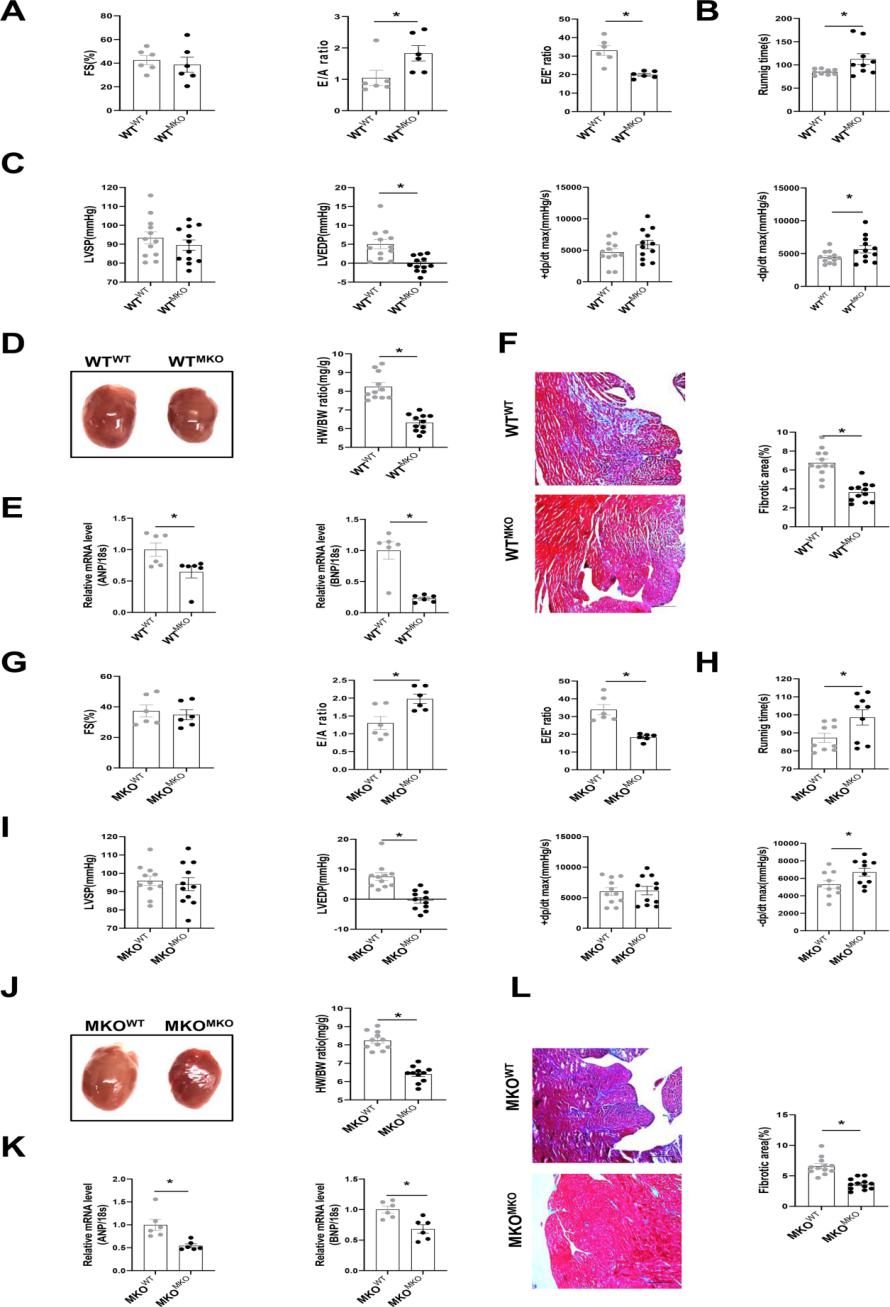
** Bone marrow-derived CXCR4-deficient cells prevent SAUNA-induced cardiac hypertrophy, fibrosis, and diastolic dysfunction. (A)** Representative left ventricular M-mode echocardiographic, pulsed-wave Doppler and tissue Doppler tracings, and measurement of FS%, E/A, and E/E' in WT^WT^ and WT^MKO^ mice after SAUNA exposure. WT^WT^, n=6; WT^MKO^, n=6. **(B)** Recording of running times during the exercise exhaustion test in WT^WT^ and WT^MKO^ mice after SAUNA exposure. WT^WT^, n=9; WT^MKO^, n=9. **(C)** LVSP, LVEDP, +dp/dt, and -dp/dt were detected in WT^WT^ and WT^MKO^ mice after SAUNA exposure. WT^WT^, n=12; WT^MKO^, n=12. **(D)** Representative heart size and HW/BW in WT^WT^ and WT^MKO^ mice after SAUNA exposure. WT^WT^, n=12; WT^MKO^, n=12. **(E)** QPCR analysis of ANP and BNP in the heart of WT^WT^ and WT^MKO^ mice after SAUNA exposure. WT^WT^, n=6; WT^MKO^, n=6. **(F)** Masson's trichrome staining of heart tissues in WT^WT^ and WT^MKO^ mice after SAUNA exposure. Scale bars =200µm. WT^WT^, n=6; WT^MKO^, n=6. **(G)** Representative left ventricular M-mode echocardiographic, pulsed-wave Doppler and tissue Doppler tracings, and measurement of FS%, E/A, and E/E' in MKO^WT^ and MKO^MKO^ mice after SAUNA exposure. MKO^WT^, n=6; MKO^WT^, n=6. **(H)** Recording of running times during the exercise exhaustion test in MKO^WT^ and MKO^MKO^ mice after SAUNA exposure. MKO^WT^, n=9; MKO^WT^, n=9. **(I)** LVESP, LVEDP, +dp/dt, and -dp/dt were detected in MKO^WT^ and MKO^MKO^ mice after SAUNA exposure. MKO^WT^, n=12; MKO^MKO^, n=12. **(J)** Representative heart size and HW/BW in MKO^WT^ and MKO^MKO^ mice after SAUNA exposure. MKO^WT^, n=12; MKO^MKO^, n=12. **(K)**QPCR analysis of ANP and BNP in the heart of MKO^WT^ and MKO^MKO^ mice after SAUNA exposure. MKO^WT^, n=6; MKO^MKO^, n=6. **(L)** Masson's trichrome staining of heart tissues in MKO^WT^ and MKO^MKO^ mice after SAUNA exposure. Scale bars =200µm. MKO^WT^, n=6; MKO^MKO^, n=6. WT^WT^, WT mice transplanted with WT bone marrow cells; WT^MKO^, WT mice transplanted with MKO bone marrow cells; MKO^WT^, MKO mice transplanted with WT bone marrow cells; MKO^MKO^, MKO mice transplanted with MKO bone marrow cells. All data were analyzed using unpaired two-tailed student's t-test.*, p<0.05.

To further explore whether CXCR4+ myeloid cells directly affect cardiac fibrosis and diastolic function, we transplanted bone marrow cells obtained from MKO mice into WT mice (WT^MKO^) and vice versa (MKO^WT^, MKO mice transplanted with WT bone marrow cells), WT^WT^ (WT mice transplanted with WT bone marrow cells) and MKO^MKO^ (MKO mice transplanted with MKO bone marrow cells) mice served as the controls (Figure S6A). CXCR4 was markedly knockdown in bone marrow cells from mice reconstituted with MKO bone marrow (Figure S6B). Baseline cardiac function, including FS%, E/A, and E/E' ratio, showed no difference among groups after bone marrow transplantation (Figure S7). After 30 days of SAUNA exposure, the FS% did not differ between WT^MKO^ mice and WT^WT^ mice (Figure 3A). As expected, an improvement in cardiac diastolic function indicated by the increased E/A ratio, reduced E/E' ratio (Figure 3A), increased exercise tolerance (Figure 3B), down-regulated LVEDP, and upregulated -dp/dt (Figure 3C) was observed in WT^MKO^ mice relative to WT^WT^ mice. In addition, cardiac hypertrophy (heart size, HW/BW ratio, and the mRNA expression of ANP and BNP)(Figure 3D and 3E) and fibrosis (Figure 3F) were all significantly reduced in heart of WT^MKO^ mice. Similar improved outcomes were also observed in MKO^MKO^ mice as compared with MKO^WT^ mice (Figure 3G-L). All these data suggest that bone marrow-derived CXCR4 cells predominantly contribute to the diastolic dysfunction in SAUNA-induced HFpEF.

### Absence of CXCR4 Inhibits Cardiac Macrophage Infiltration and heart inflammation response

**Figure 4:**
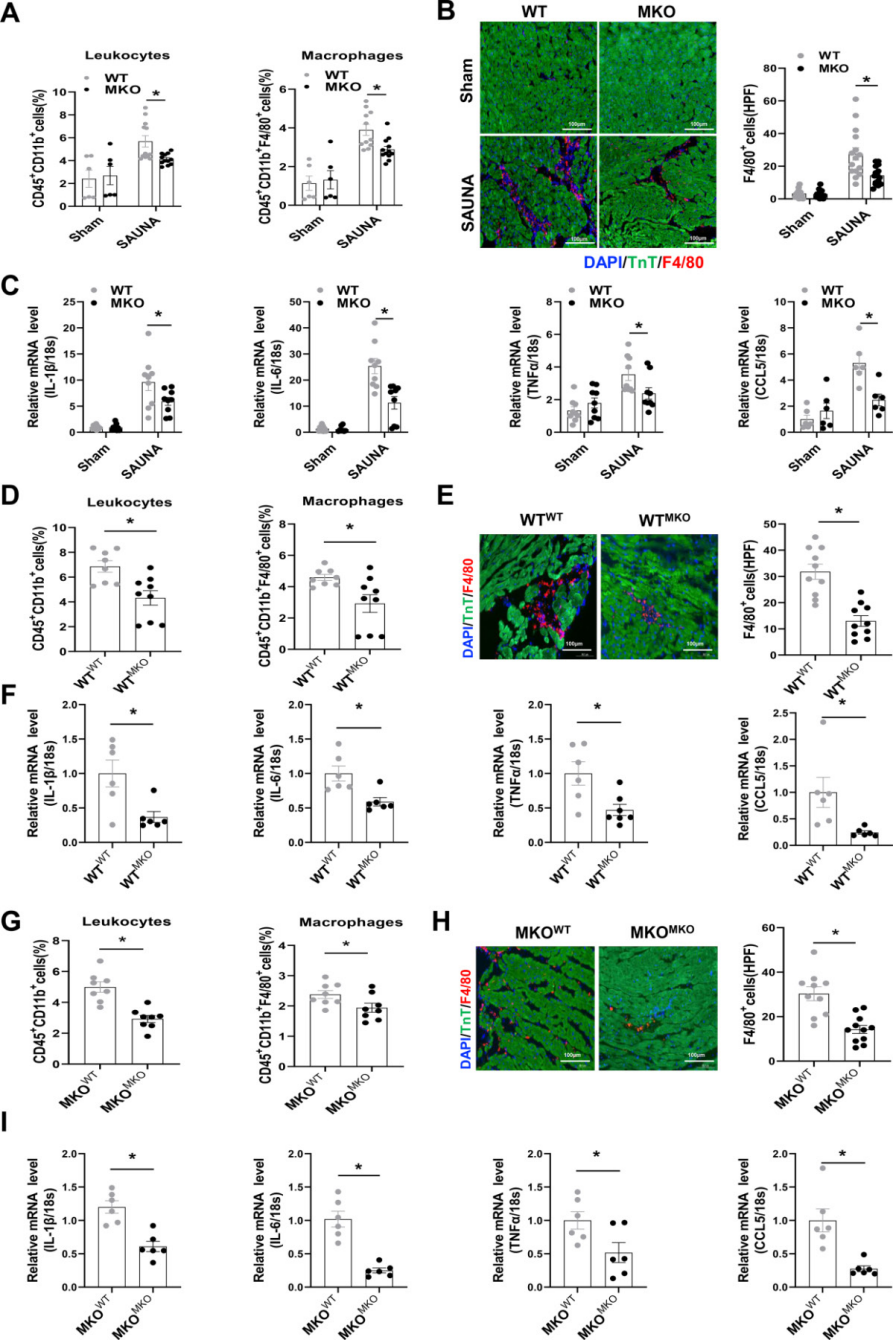
** Myeloid-specific CXCR4 knockout mice are protected from SAUNA-induced cardiac macrophage infiltration and inflammation response. (A)** Flow cytometry analysis of CD45+CD11b+leukocytes and CD45+CD11bF4/80+macrophages in the heart of WT mice and MKO mice after sham or SAUNA operation. WT^sham^, n=6; MKO^sham^, n=6; WT^SAUNA^, n=11; MKO^SAUNA^, n=12. **(B)** Immunofluorescence staining of F4/80 (red) and anti-Troponin T antibody (green) (DAPI, blue) in in the heart of WT mice and MKO mice after sham or SAUNA operation, respectively, Scale bar = 100μm. WT^sham^, n=6; MKO^sham^, n=6; WT^SAUNA^, n=11; MKO^SAUNA^, n=12. **(C)**QPCR analysis of IL-1β, IL-6, TNFα, and CCL5 in WT and MKO heart. WT^sham^, n=9; MKO^sham^, n=9; WT^SAUNA^, n=9; MKO^SAUNA^, n=9. **(D)** Flow cytometry analysis of CD45+CD11b+leukocytes and CD45+CD11b+F4/80+macrophages in the heart of WT^WT^ and WT^MKO^ mice after SAUNA exposure. WT^WT^, n=8; WT^MKO^, n=9. **(E)** Immunofluorescence staining of F4/80 (red) and anti-Troponin T antibody (green) (DAPI, blue) in the heart of WT^WT^ and WT^MKO^ mice after SAUNA exposure. respectively, Scale bar = 100μm. WT^WT^, n=6; WT^MKO^, n=6. **(F)** QPCR analysis of IL-1β, IL-6, TNFα, and CCL5 in the heart of WT^WT^ and WT^MKO^ mice after SAUNA exposure. **(G)** Flow cytometry analysis of CD45+CD11b+leukocytes and CD45+CD11b+F4/80+macrophages in the heart of MKO^WT^ and MKO^MKO^ mice after SAUNA exposure. MKO^WT^, n=8; MKO^MKO^, n=8. **(H)** Immunofluorescence staining of F4/80 (red) and anti-Troponin T antibody (green) (DAPI, blue) in the heart of MKO^WT^ and MKO^MKO^ mice after SAUNA exposure. respectively, Scale bar = 100μm. MKO^WT^, n=6; MKO^MKO^, n=6. **(I)** QPCR analysis of IL-1β, IL-6, TNFα, and CCL5 in the heart of MKO^WT^ and MKO^MKO^ mice after SAUNA exposure. MKO^WT^, n=6; MKO^MKO^, n=6. IL-1β, interleukin-1β; TNFα, tumor necrosis factor α; CCL5, C-C Motif Chemokine Ligand 5. Data were analyzed using two-way ANOVA with Bonferroni's multiple comparisons test **(A-C)** and unpaired two-tailed student's t-test **(D-I)**. *, p<0.05.

Macrophage chemotaxis and migration are essential during the progression of inflammation-immune response and cardiac remodeling 25. Flow cytometry analysis indicated that the absence of CXCR4 inhibited the infiltration of leukocytes, especially macrophages, in the heart after SAUNA exposure (Figure 4A). Immunofluorescence staining further confirmed that deletion of CXCR4 in myeloid cells significantly abrogated SAUNA-induced macrophage recruitment (Figure 4B). Additionally, the hearts of MKO mice had fewer pro-inflammatory cytokines, including interleukin-1β(IL-1β), IL-6, tumor necrosis factor α (TNFα), and C-C Motif Chemokine Ligand 5 (CCL5) (Figure 4C). In bone marrow transplantation experiments, WT^MKO^ mice exhibited reductions in infiltration of leukocytes and macrophages (Figure 4D-E), as well as the proinflammatory cytokines (Figure 4F) compared to WT^WT^ mice. Similar results (less macrophage infiltration and fewer inflammatory cytokines) were also observed in MKO^MKO^ mice compared with MKO^WT^ mice (Figure 4G-I). Collectively, these data indicated that CXCR4 mediates the accumulation of macrophages which may further contribute to cardiac inflammation and diastolic dysfunction.

### CXCR4 governs a pro-inflammatory phenotype in macrophages

As ligands for CXCR4, chemokine (C-X-C motif) ligand 12 (CXCL12) and high mobility group box-1 protein (HMGB1), but not macrophage migration-inhibitory factor*s (MIF) were* upregulated in SAUNA-exposed heart compared to sham group (Figure S8). Current studies have shown that HMGB1 promotes the recruitment of inflammatory cells to damaged tissues by forming a complex with CXCL12 and signaling via CXCR4 26. Also, a basal level of CXCL12 is available in the medium of macrophage, which could be significantly increased under HMGB1 stimulation 26, 27. In this study, we also observed that HMGB1 induces CXCL12 transcription (Figure S9A). Consistently, we found that production of pro-inflammatory cytokines from macrophages could be triggered by HMGB1 alone or in complex with CXCL12 (Figure S9B).

**Figure 5:**
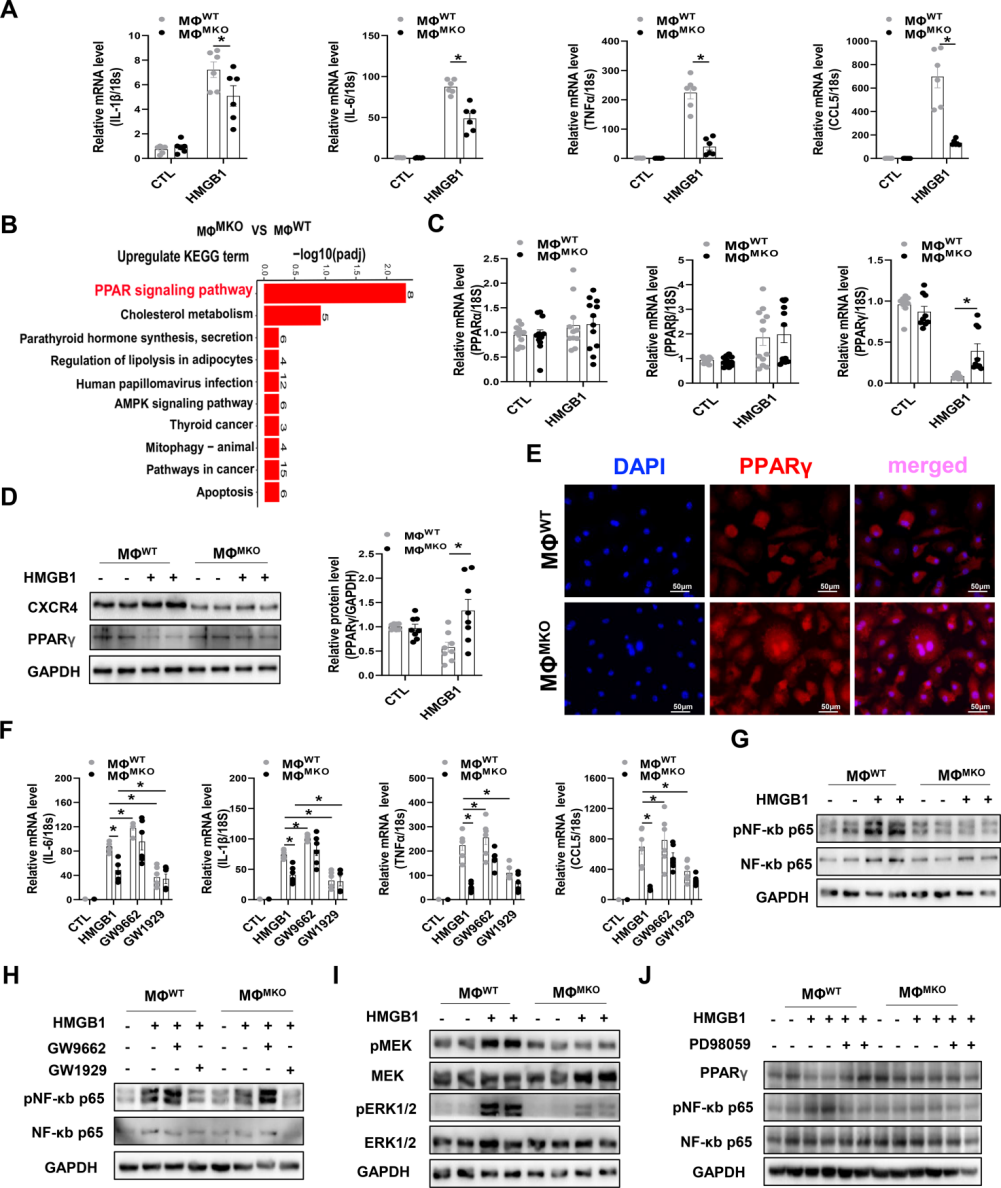
** CXCR4 governs a pro-inflammatory phenotype in macrophages. (A)** QPCR analysis of CXCR4, IL-1β, IL-6, TNFα, and CCL5 in MΦ^WT^ and MΦ^MKO^ after HMGB1 treatment.** (B)** The KEGG pathway analysis of cluster in upregulated gene terms of MΦ^WT^ and MΦ^MKO^ after HMGB1 treatment. Top 10 of enriched KEGG term are listed. **(C)** QPCR analysis of PPARα, PPARβ, and PPARγ in MΦ^WT^ and MΦ^MKO^ with or without HMGB1 treatment. **(D)** Western blot analysis of CXCR4 and PPARγ in MΦ^WT^ and MΦ^MKO^ after HMGB1 treatment. **(E)** Immunofluorescence staining of PPARγ (red) (DAPI, blue) in MΦ^WT^ and MΦ^MKO^ after HMGB1 treatment. Scale bar = 50μm.** (F)** QPCR analysis of IL-1β, IL-6, TNFα, and CCL5 in MΦ^WT^ and MΦ^MKO^ with GW9662 or GW1929 after HMGB1 treatment.** (G)** Western blot analysis of pNF-κB p65 and NF-κB p65 in MΦ^WT^ and MΦ^MKO^ after HMGB1 treatment. **(H)** Western blot analysis of pNF-κB p65 and NF-κB p65 in MΦ^WT^ and MΦ^MKO^ with GW9662 or GW1929 after HMGB1 treatment.** (I)** Western blot analysis of pMEK, MEK, pERK1/2 and ERK1/2 in MΦ^WT^ and MΦ^MKO^ after HMGB1 treatment. **(H)** Western blot analysis of pNF-κB p65 and NF-κB p65 in MΦ^WT^ and MΦ^MKO^ withPD98095 after HMGB1 treatment. GO, Gene Ontology; KEGG, Kyoto Encyclopedia of Genes and Genomes; All data were analyzed using two-way ANOVA with Bonferroni's multiple comparisons test. *, p<0.05.

An RNA-sequencing (RNA-seq) was performed in macrophages derived from WT or MKO mice (MΦ^WT^ or MΦ^MKO^) treated with either HMGB1 or vehicle. The downregulated GO term analysis of MΦ^MKO^ revealed that the regulation of inflammatory response was the most predominant functional category in the biological processes (BP) (Figure S10A). Further circos plots depicted the differential genes associated with the regulation of inflammatory response in MΦ^MKO^ (Figure S10B). This finding was further supported by diminished expression of pro-inflammatory cytokines, including IL-1β, IL-6, TNFα, and CCL5 in MΦ^MKO^ in the presence of HMGB1 compared to MΦ^WT^ (Figure 5A), which was consistent with the blunted pro-inflammatory cytokines in MKO mice compared to WT mice after SAUNA exposure (Figure 4C). Moreover, the kyoto encyclopedia of genes and genomes (KEGG) enrichment analysis indicated that the peroxisome proliferator-activated receptor (PPAR) signaling pathway might be involved in CXCR4 regulated-cytokine production (Figure 5B). Instead of PPARα and PPARβ, PPARγ from the PPAR family showed a significant upregulated expression level in MΦ^MKO^ compared to MΦ^WT^ after HMGB1 treatment (Figure 5C-D). In addition, silencing of CXCR4 induces more nuclear PPARγ localization (Figure 5E), acting as a vital negatively transcriptional regulator of pro-inflammatory cytokines 28. When PPARγ activity was promoted by agonist GW1929, the expression of IL-1β, IL-6, TNFα, and CCL5 was downregulated. On the other hand, the upregulated expression of IL-1β, IL-6, TNFα, and CCL5 was attributed to the presence of PPARγ antagonist GW9662 (Figure 5F). In addition, CXCR4 blockade downregulated the expression of NF-κb in response to HMGB1 (Figure 5G). PPARγ is known for modulating pro-inflammatory cytokine production through the NFkB signaling pathway 29, 30. Correspondingly, our results showed that the PPARγ appeared to nagetively regulate the phosphorylation of NF-κB p65 (Figure 5H). Interestingly, PPARγ is a direct target of ERK, which may result in decreased PPARγ transcriptional activity 31. Herein, CXCR4 knockdown significantly repressed the phosphorylation of MEK and ERK after HMGB1 treatment (Figure 5I). As expected, Erk inhibitor (PD98059) enhanced PPARγ expression and suppressed NF-κB p65 phosphorylation (Figure 5J). Collectively, CXCR4 promoted cytokine secretion from macrophages by disrupting PPARγ activity.

### Activation of the CXCL3-CXCR2 confers the fibroblast to myofibroblast transition in a CXCR4 -dependent way

**Figure 6:**
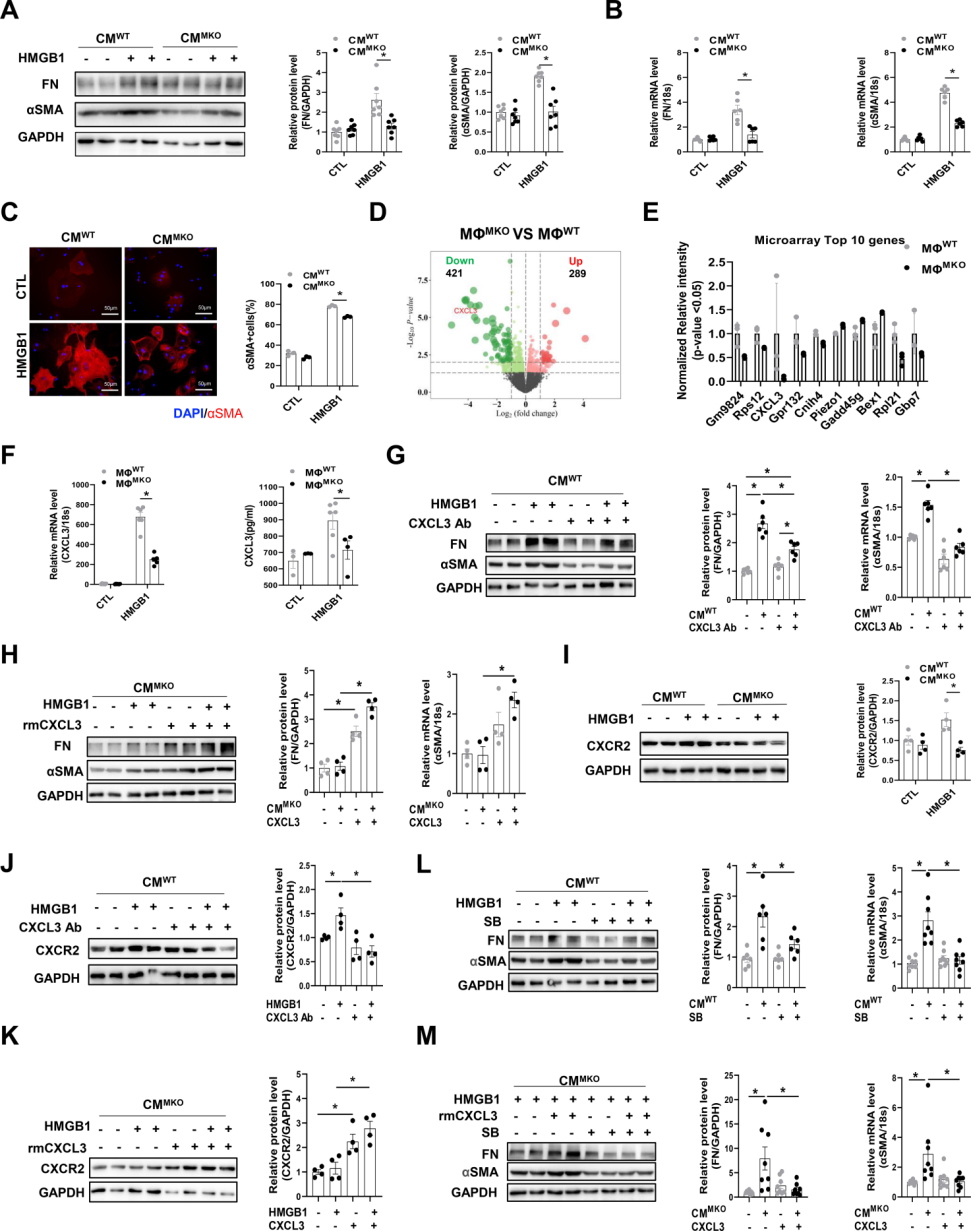
** CXCR4-deficient macrophages attenuate myofibroblast differentiation via CXCL3-CXCR2 axis. (A)** Western blot analysis of FN and αSMA in CFs co-cultured in MΦ^WT^ and MΦ^MKO^-CM with or without HMGB1 treatment.** (B)** QPCR analysis of FN and αSMA in CFs co-cultured in MΦ^WT^ and MΦ^MKO^-CM with or without HMGB1 treatment. **(C)** Immunofluorescence staining of αSMA+ cells in CFs co-cultured in MΦ^WT^ and MΦ^MKO^-CM with or without HMGB1 treatment. (αSMA. red; DAPI. blue), Scale bar = 50μm. **(D)** Volcano plot of RNA sequencing MΦ^WT^ and MΦ^MKO^ after HMGB1 treatment.** (E)** RNA array analysis of the indicated genes in MΦ^WT^ and MΦ^MKO^ after HMGB1 treatment. **(F)** QPCR and elisa analysis of CXCL3 MΦ^WT^ and MΦ^MKO^-CM with or without HMGB1 treatment. **(G)** Western blot analysis of FN and αSMA in CFs co-cultured in MΦ^WT^-CM after HMGB1 treatment with or without CXCL3 Ab. **(H)** Western blot analysis of FN and αSMA in CFs co-cultured in MΦ^MKO^-CM after HMGB1 treatment with or without rmCXCL3 protein.** (I)** Western blot analysis of CXCR2 in CFs co-cultured in MΦ^WT^ and MΦ^MKO^-CM with or without HMGB1 treatment. **(J)** Western blot analysis of CXCR2 in CFs co-cultured in MΦ^WT^-CM after HMGB1 treatment with or without CXCL3 Ab.** (K)** Western blot analysis of CXCR2 in CFs co-cultured in MΦ^MKO^-CM after HMGB1 treatment with or without rmCXCL3. **(L)**Western blot analysis of FN and αSMA in CFs co-cultured in MΦ^WT^ -CM after HMGB1 treatment with or without SB225002. **(M)**Western blot analysis o FN and αSMA in CFs co-cultured in MΦ^MKO^-CM after HMGB1 treatment with or without rmCXCL3 or SB225002. FN, fibronectin; αSMA, a-smooth muscle actin; CFs, cardiac fibroblasts; MΦ^WT^ macrophages derived from the bone marrow WT mice; MΦ^MKO^, macrophages derived from the bone marrow MKO mice. CM, conditioned medium. CXCL3, Chemokine (C‑X‑C) motif ligand 3. All data were analyzed using two-way ANOVA with Bonferroni's multiple comparisons test. *, p<0.05.

Activated fibroblasts and myofibroblasts are the central cellular effectors in cardiac fibrosis, serving as the primary source of matrix proteins 32. To test whether CXCR4+macrophages directly influenced myofibroblast differentiation* in vitro*. The conditioned medium (CM) was collected from MΦ^WT^ or MΦ^MKO^ treated with either HMGB1 or vehicle and then added to cardiac fibroblasts (CFs) isolated from WT neonatal mice. Fibronectin (FN) and α-smooth muscle actin (αSMA) were used as indicators of CFs activation. The expression of FN and αSMA was significantly amplified in CFs co-cultured in MΦ^WT^-CM with HMGB1 (Figure 6A and 6B). The result is further supported by immunofluorescence labeling of αSMA (Figure 6C). Moreover, CFs co-cultured in medium from MΦ^MKO^ exhibited impaired synthesis of the extracellular matrix, including the collagen and fibronectin (COL3A1, COL4A1, COL5A1, and FN1), several proteoglycans (cTGF and SPARC), cell-matrix focal adhesion molecules (ITGA4 and ITGB5), matrix metalloproteinases (MMPs), and their endogenous inhibitors (TIMPs) such as Timp2 and Timp3 (Figure S11). Diminished myofibroblast differentiation (Figure S12A) and reduced ECM (Figure S12B) were further validated in the heart of SAUNA-exposed MKO mice as compared with WT mice. RNA-seq revealed a significantly different profile between MΦ^MKO^ and MΦ^WT^ under HMGB1 stimulation (Figure 6D). Notably, CXCL3 was identified as one of the top down-regulated genes (Figure 6E and Table S2). Immunofluorescence staining validated that CXCL3 localized predominately with macrophages in SAUNA-exposed heart (Figure S13). QPCR assay and ELISA further confirmed the expression of CXCL3 was reduced in HMGB1-stimulated MΦ^MKO^ compared to MΦ^WT^ (Figure 6F). Similarly, a corresponding decrease of CXCL3 was observed in the hearts of MKO mice, WT^MKO^ mice, and MKO^MKO^ mice compared with their respective controls after SAUNA exposure (Figure S14A-B). The activation of the fibroblasts could be partly blocked by adding an anti-CXCL3 neutralizing antibody (CXCL3 Ab) to CM derived from MΦ^WT^ reflected by reduced FN and αSMA (Figure 6G). Reciprocally, MΦ^MKO^-mediated myofibroblast differentiation was further supported by increased αSMA and FN upon the addition of recombinant mouse CXCL3 protein (rmCXCL3) to CM (Figure 6H). Consistently, we also found that CXCL3 clustered in αSMA-positive areas in the heart of WT^SAUNA^ mice (Figure S14C).

Moreover, the expression of CXCR2 (the receptor of CXCL3) was decreased in CFs co-cultured in MΦ^MKO^-CM compared to CFs co-cultured in MΦ^WT^-CM (Figure 6I). A similar reduction of CXCR2 was also observed in the hearts of MKO mice, WT^MKO^ mice, and MKO^MKO^ mice compared with their respective controls in response to SAUNA (Figure S15A-B). Immunofluorescence staining showed that CXCR2 expression was largely expressed on α-SMA+myofibroblasts (Figure S15C). Further, CXCL3 Ab could counteract the upregulated CXCR2 in CFs cultured in MΦ^WT^-CM (Figure 6J), and the down-regulated CXCR2 could be enhanced by adding rmCXCL3 into CFs cultured in MΦ^MKO^-CM (Figure 6K). Moreover, the CXCR2 inhibitor SB225002 at least in part attenuated the activation of the fibroblasts treated with MΦ^WT^-CM or rmCXCL3 protein (Figure 6L and 6M). The data indicated that CXCR4 significantly augmented CXCL3 expression, which at least partly contributed to fibrosis by promoting myofibroblast differentiation.

### Circulatory CXCR4^+^ inflammatory cells are increased in patients with HFpEF

**Figure 7:**
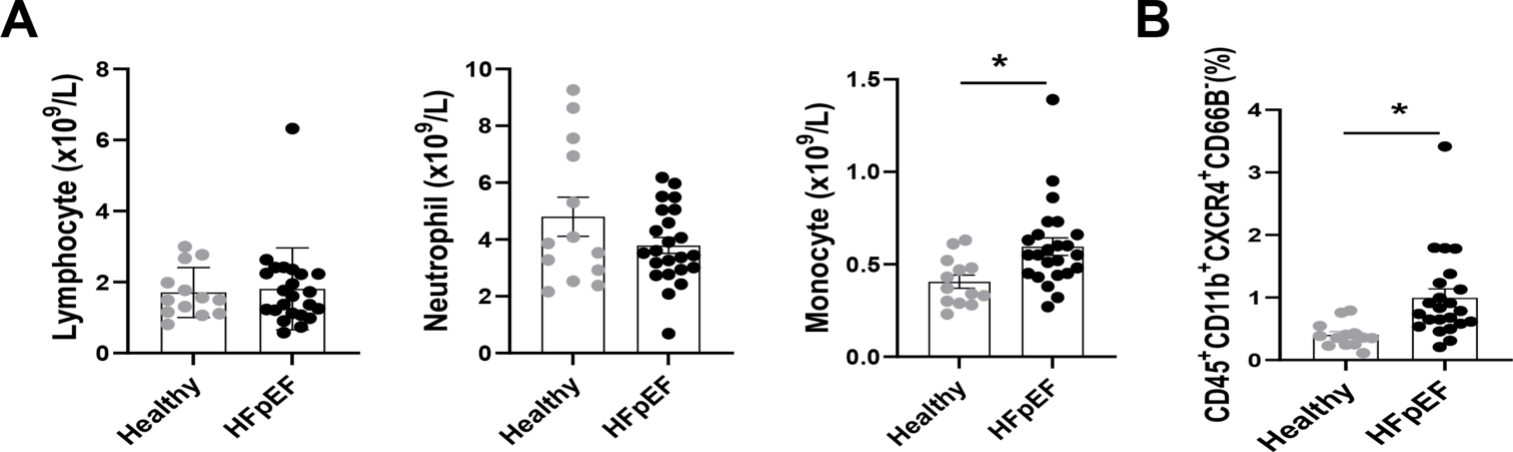
** Circulatory CXCR4^+^ inflammatory cells are increased in patients with HFpEF. (A)** Number of neutrophils, monocytes, and lymphocytes in blood obtained from healthy individuals and HFpEF patients. Healthy individuals, n =13; HFpEF patients, n = 23. **(B)** Flow cytometry analysis of CD45+ CD11b+ CD66B-CXCR4+ cells in blood obtained from healthy individuals and HFpEF patients. Healthy individuals, n =13; HFpEF patients, n = 23. All data were analyzed using unpaired two-tailed student's t-test. * p<0.05.

The relationship between CXCR4-expressed macrophages and HFpEF was further validated in a clinical study, in which 23 patients with HFpEF and 13 healthy individuals were included. The baseline characteristics of HFpEF patients and healthy individuals are shown in Table S3. The blood routine showed an increased density of blood monocytes in HFpEF patients, whereas neutrophils and lymphocyte numbers were unchanged (Figure 7A). Besides, flow cytometry analysis (Figure S16) revealed an increase of CXCR4+monocytes in HFpEF patients compared to healthy individuals (Figure 7B).

## Discussion

Previous studies have suggested that the evolving view of HFpEF as a chronic inflammatory condition is promoted by the recruitment of monocytes in the heart and boosting pro-inflammatory cytokines, resulting in myocardial fibrosis and diastolic dysfunction 33, 34. First, our study may be clinically relevant because the circulatory CXCR4+ inflammatory cells, mainly monocytes, were markedly elevated in HFpEF patients. Second, during the progression of HFpEF, macrophages but not neutrophils represent the main infiltrating inflammatory cells in the heart, abundantly expressing CXCR4. Third, myeloid-specific CXCR4 deficiency impeded macrophage infiltration and inflammatory response in HEpEF heart tissue, thereby ameliorating cardiac fibrosis as well as improving cardiac diastolic function. Fourth, CXCR4 augmented the pro-inflammatory state in macrophage via repressing PPARγ activity. In the last part, the role of CXCR4-expressing macrophages on myofibroblast differentiation is at least partly attributed to CXCL3.

CXCR4 mediates the mobilization, recruitment, and retention of resting leukocytes during many physiological processes 35. Recent studies have reported that CXCR4 functioned a pivotal regulator that facilitates leukocytes recruitment at inflammation sites 19, 35. In this study, CXCR4 was highly expressed in macrophages infiltrated in the SAUNA-induced heart. Furthermore, myeloid-specific CXCR4 deficiency significantly attenuated the infiltration of macrophages, weakened the inflammation response, and mitigated cardiac diastolic dysfunction. Also, similar results were found in chimeric mice created by the bone marrow transplantation experiment. These data implied that CXCR4 is crucial for macrophage infiltration, inflammation response, and cardiac dysfunction in response to SAUNA. Although this study emphasized the novel and critical role of CXCR4 in macrophages in pathological processes during HFpEF, we cannot exclude the possibility that CXCR4 regulates the function of other immune cells in HFpEF development. Additional mechanistic studies are needed to understand the impact of CXCR4 on other immune cells fully.

Several studies have reported that diastolic dysfunction occurs in response to long-term cardiac overload, which is frequently aligned with excessive inflammation overreaction and myocardial fibrosis excesssion 12, 36-38. Cardiac fibrosis is a common pathological consequence of excessive inflammation. For example, IL-6 and cytokine signaling 1 (Socs1), classic pro-inflammatory cytokines, bring on myofibroblasts' overabundance and excessive extracellular matrix deposition 25, 33, 39-41. According to this, blocking inflammatory cell infiltration and inflammatory secretion may attenuate fibrosis and improve diastolic dysfunction. However, the mechanisms how macrophages stimulate fibroblast differentiation into myofibroblast to drive cardiac fibrosis have not yet been elucidated completely. Herein, transcriptomic profiling data revealed that CXCR4 governed pro-inflammatory transcriptional signature in macrophages and produced more pro-inflammatory cytokines such as IL-1β, IL-6, TNFα, and CCL5, all of which are well-established cytokines involving in cardiac fibrosis 32, 42. Notably, CXCL3 was singled out as one of the top down-regulated genes in MΦ^MKO^ compared to MΦ^WT^, which has been previously reported to recruit inflammatory cells 43. This finding may partly account for the blunted macrophage infiltration in the heart of MKO mice after SAUNA exposure. Interestingly, in the SAUNA-induced heart, CXCL3 was mainly clustered in αSMA-positive areas. Meanwhile, its corresponding receptor CXCR2 was relatively expressed on fibroblasts. For the first time, we demonstrate that activation of the CXCL3-CXCR2 axis promotes the differentiation of cardiac fibroblasts into myofibroblasts. Also, attenuated fibrosis in MKO mice was at least partly attributed to the reduction of CXCL3. Taken together, these lines of evidence suggest that CXCR4 ablation in macrophages exert suppressive effects on the expression of pro-inflammatory genes, including IL-1β, IL-6, TNFα, CCL5, and CXCL3, thereby alleviating fibrosis in HFpEF.

PPARs (α, δ, and γ), are ligand-activated nuclear receptors, regulating various metabolic processes such as cell proliferation, differentiation, and metabolism 44, 45. The KEGG enrichment analysis in the study indicated that PPAR was the most significant upregulated signaling pathway in MΦ^MKO^ compared to MΦ^WT^. Instead of PPARα and PPARβ, PPARγ from the PPAR family was augmented in MΦ^MKO^ compared to MΦ^WT^. Moreover, macrophage PPARγ activity has been proved to be pivotal in moderating inflammation response 46-50. Our study demonstrated that CXCR4 activated the pro-inflammatory phenotype of macrophages through restraining PPARγ expression. Conversely, in the absence of CXCR4, macrophages tended to exert greater effects on enhancing PPARγ activity, leading to less pro-inflammatory cytokines, thus directly accounting for the attenuated inflammatory response in the heart of MKO post SAUNA. However, an additional in-depth research is required to elucidate the exact mechanisms by which CXCR4 regulates PPARγ activity.

In conclusion, our data revealed that the CXCR4 orchestrates a pro-inflammatory phenotype in macrophages by repressing PPARγ activity, thereby aggravating inflammatory response, myocardial fibrosis, and cardiac dysfunction. These effects could be reversed by myeloid-specific CXCR4 deficiency (Figure 7). Therefore, CXCR4 inhibition may constitute a novel therapeutic option to block macrophage CXCR4 signaling to prevent cardiac diastolic dysfunction in patients with HFpEF.

## Supplementary Material

Supplementary figures and tables.

## Abbreviations

HFpEF: Heart failure with preserved ejection fraction
CXCR: CXC chemokine receptor
WT: Wild-type
MKO: Myeloid-specific CXCR4 knockout mice
MΦ: Macrophage
CM: Conditioned medium
E wave: Early peak diastolic blood flow
A wave: Late diastolic blood flow
E' wave: Early diastolic exercise rate
A' wave: Late diastolic exercise rate
E/A: Early to late mitral inflow velocity ratio
E/E': Transmitral to mitral annular early diastolic velocity ratio
FS: Fraction shortening
LVEDP: Left ventricular end-diastolic pressure
LVSP: Left ventricular systolic pressure
+dp/dt: Maximal rate of the increase of left ventricular pressure
-dp/dt: Maximal rate of the decrease of left ventricular pressure
ANP: Atrial natriuretic peptide
BNP: Brain natriuretic peptide
HW/B: Heart weight to body weight
WGA: Wheat germ agglutinin
IL-1β: Interleukin-1β
IL-6: Interleukin-6
TNFα: Tumor necrosis factor α
HMGB1: High mobility group box-1 protein
αSMA: a-smooth muscle actin
FN: Fibronectin
CFs: Cardiac fibroblasts
CXCL12: Chemokine (C X C) motif ligand 12
MIF: macrophage migration inhibitory factor
CXCL3: Chemokine (C X C) motif ligand 3
CXCL3 Ab: Anti-CXCL3 neutralizing antibody
rmCXCL3: Recombinant mouse CXCL3 protein
SB: SB225002
PPAR: Peroxisome proliferators-activated receptor
