# Supplementary material for: CXCR4-dependent macrophage-to-fibroblast signaling contributes to cardiac diastolic dysfunction in heart failure with preserved ejection fraction
Source: Int J Biol Sci. 2022 Jan 9;18(3):1271–87. doi: 10.7150/ijbs.65802 (PMC8771855; doi:10.7150/ijbs.65802)
Supplement: Supplementary file 1 — Supplementary figures and tables. [file ijbsv18p1271s1.pdf]

Supplemental materials

Full title. CXCR4-dependent macrophage-to-fibroblast signaling contributes to cardiac diastolic dysfunction in heart failure with preserved ejection fraction.

Ning Zhang[1#], Qunchao Ma[1#], Yayu You[1], Xiangyang Xia[1], Cuiping Xie[1], Yuxue Huang[1], Zhuo Wang[1], Feiming Ye[1], Zhaosheng Yu[2], Xiaojie Xie[1*]

# NZ and QCM contributed equally to this article.

1. Department of Cardiology, Cardiovascular Key Laboratory of Zhejiang Province, Second Affiliated Hospital, Zhejiang University College of Medicine, 88 Jiefang Rd, Hangzhou, Zhejiang Province, 310009, PR China

2. State Key Laboratory of Fluid Power and Mechatronic Systems, Department of Mechanics, Zhejiang University, Hangzhou 310027, China

Running title. Macrophagic CXCR4 and HFpEF

*__Corresponding author.__

Xiaojie Xie, Ph.D., Department of Cardiology, Second Affiliated Hospital, College of Medicine, Zhejiang University, and Cardiovascular Key Laboratory of Zhejiang Province. 88 Jiefang Rd, Hangzhou 310009, P.R. China.

Tel.13705817626

E-mail address. xiexj@zju.edu.cn

Supplemental figures and figure legends

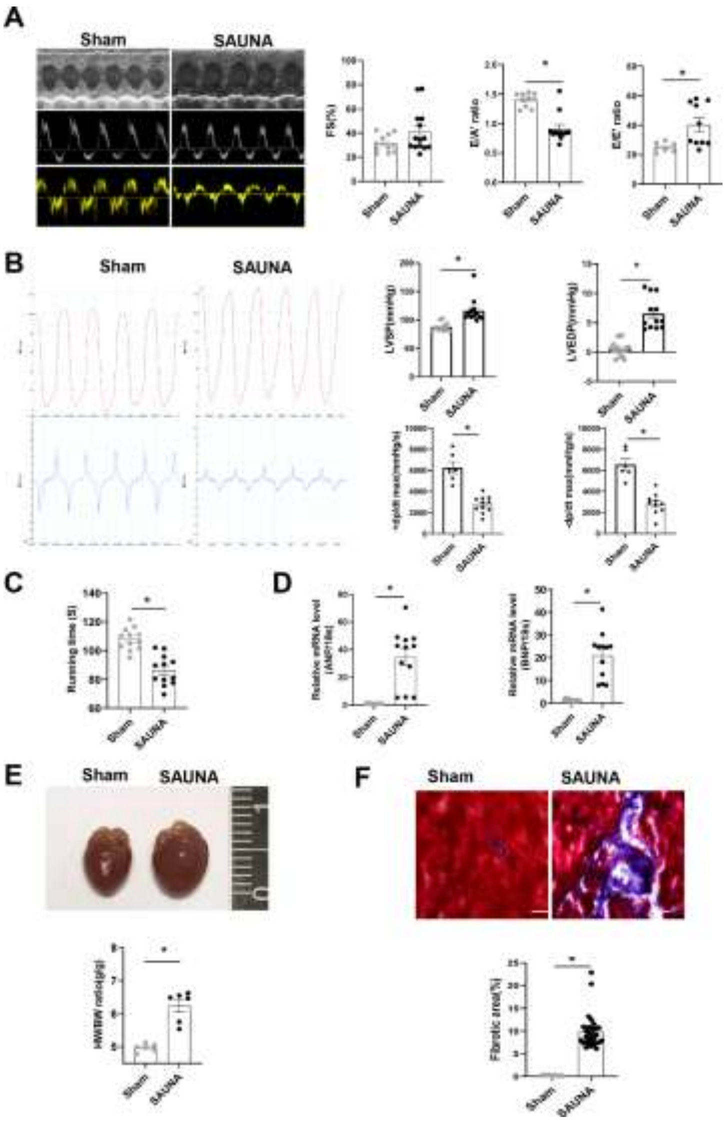

Figure S1. SAUNA induced HFpEF.

(A) Representative left ventricular M-mode echocardiographic, pulsed-wave Doppler and tissue Doppler tracings, measurement of FS%, E/A, and E/E'. **(B)** Graphic representation and quantification of LVSP and LVEDP, +dp/dt, and -dp/dt. **(C)** Recording of running times during exercise exhaustion test. **(D)** QPCR analysis of the mRNA levels of ANP, and BNP in the heart. **(E)** Representative heart size and HW/BW. **(F)** Masson's trichrome staining of heart tissues and quantification of fibrotic area. scale bars=50 µm. Sham, n = 10; SAUNA, n = 14. All data were analyzed using unpaired two-tailed student's t-test. *, $p<0.05$.

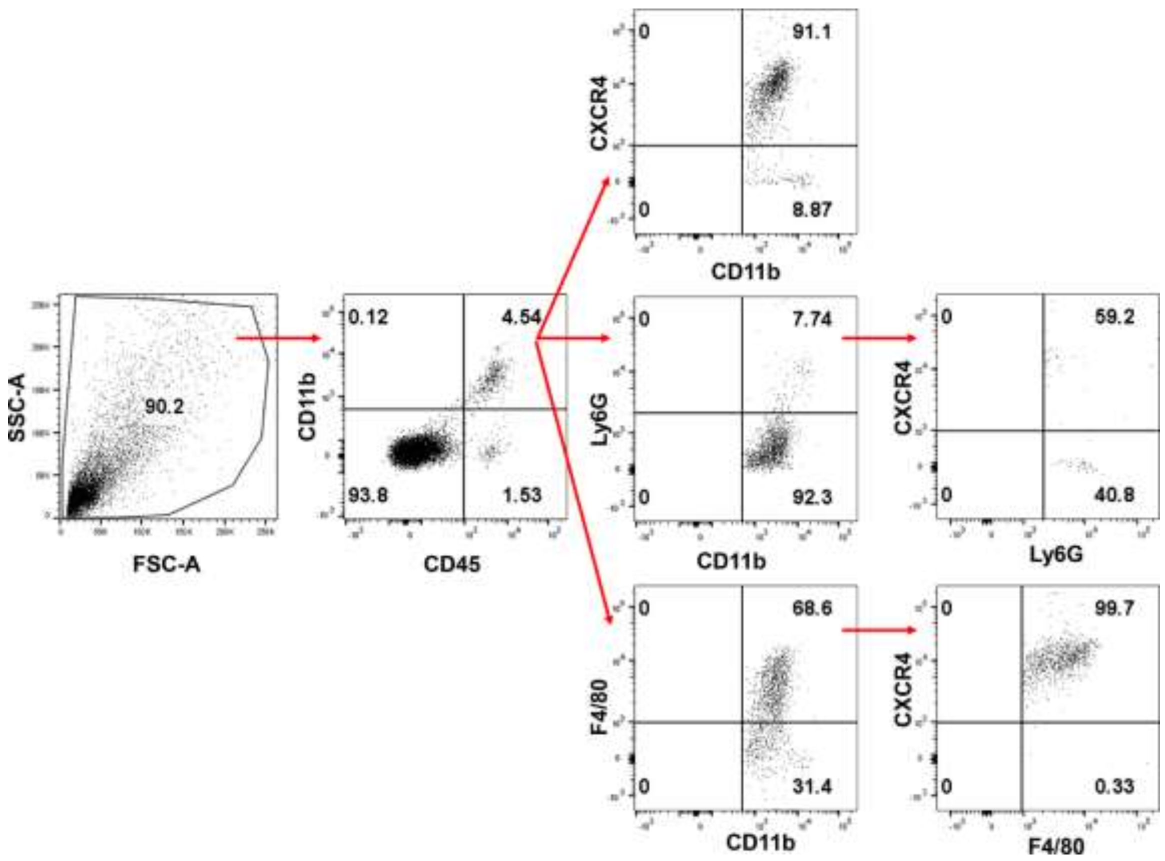

Figure S2. Gating strategy for immune cells in heart post-SAUNA

Gating strategy of CD45+ CD11b+ leukocytes and CD45+CD11b+ F4/80+ macrophages in the heart of sham and SAUNA group.

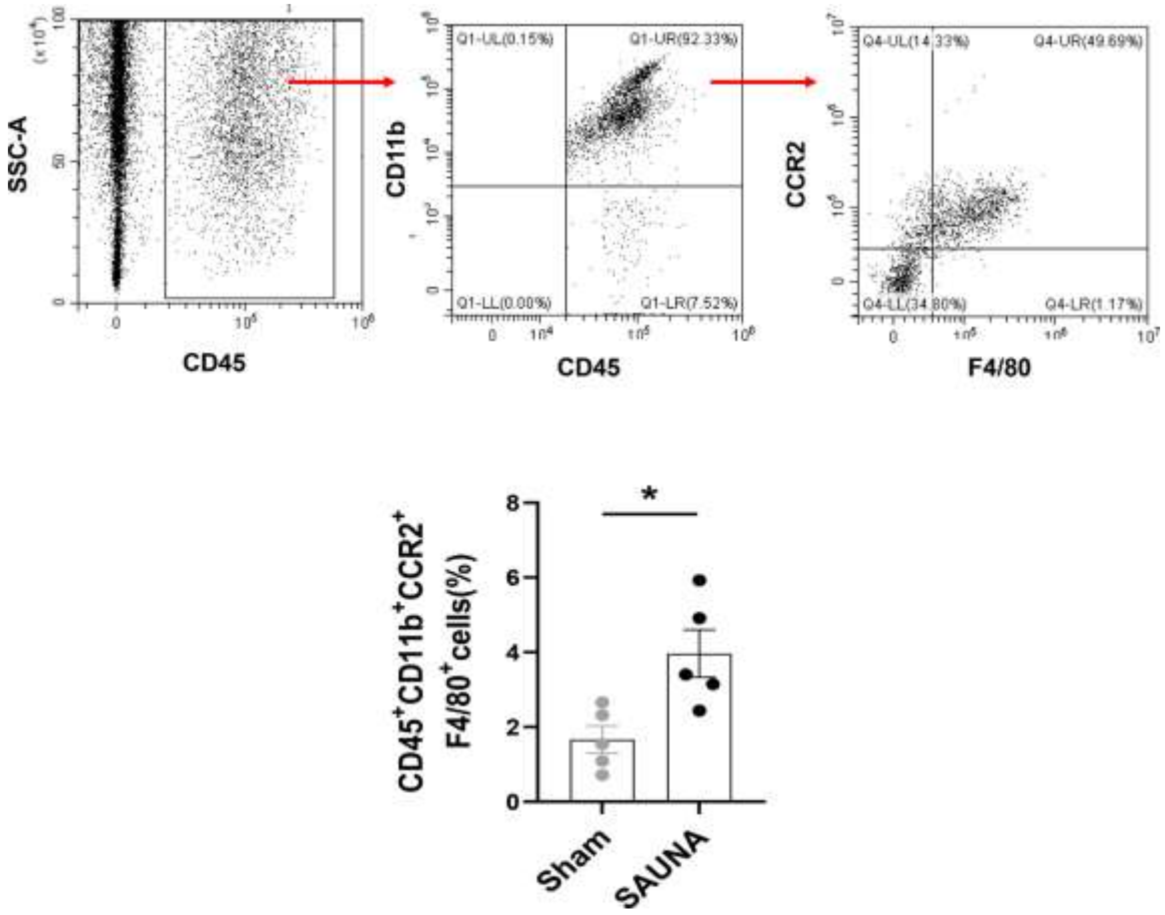

Figure S3. SAUNA induced bone marrow-derived macrophages accumulating in the heart.

Flow cytometry analysis the CD45+CD11b+CCR2+F4/80+ macrophages in the heart of sham and SAUNA group. Sham, n = 5; SAUNA, n = 5. Data were analyzed using two-way ANOVA with Bonferroni's multiple comparisons test. *, p<0.05.

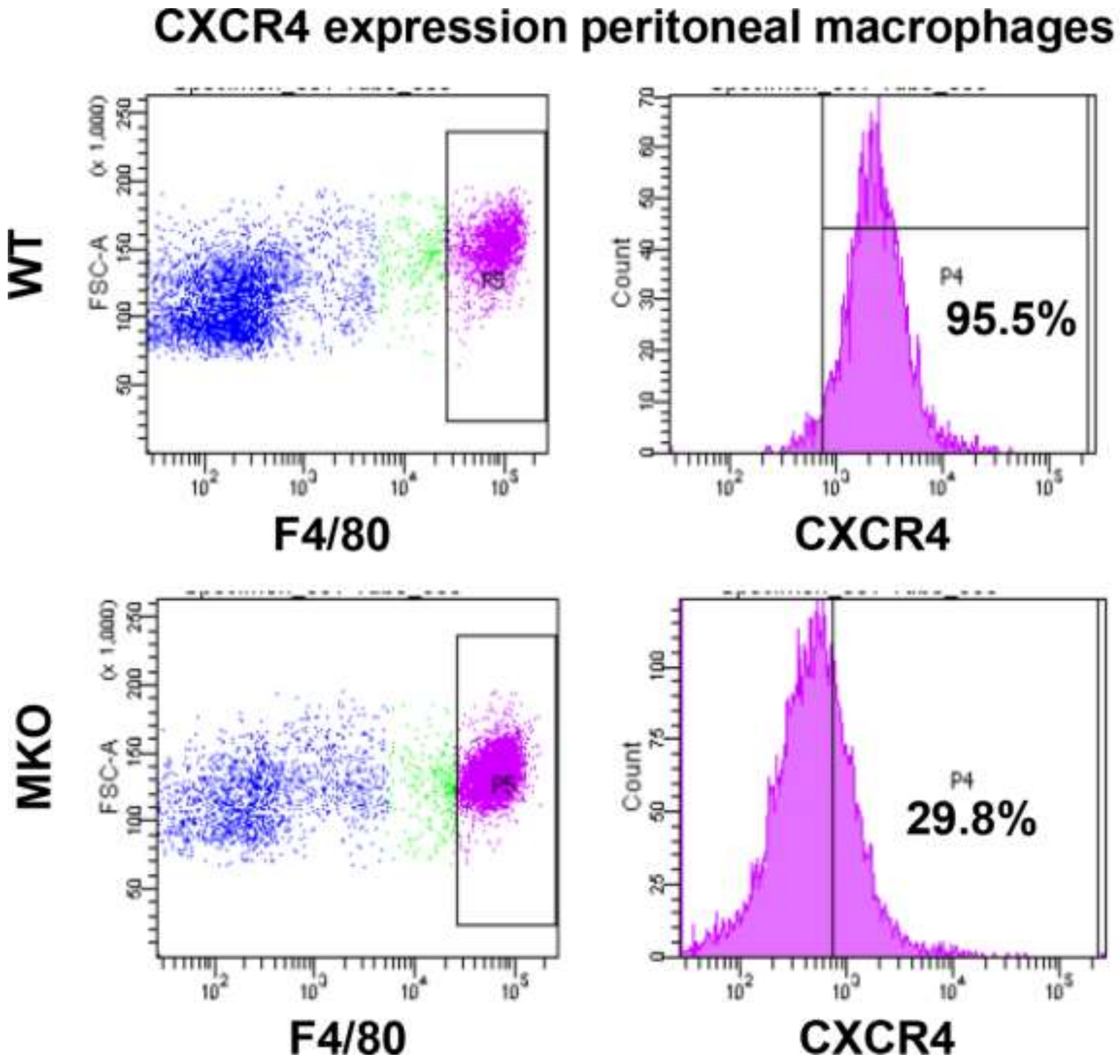

Figure S4. CXCR4 was knockout in peritoneal macrophages of MKO mice.

Flow cytometry analysis of CXCR4 expression in peritoneal macrophage of WT and MKO mice.

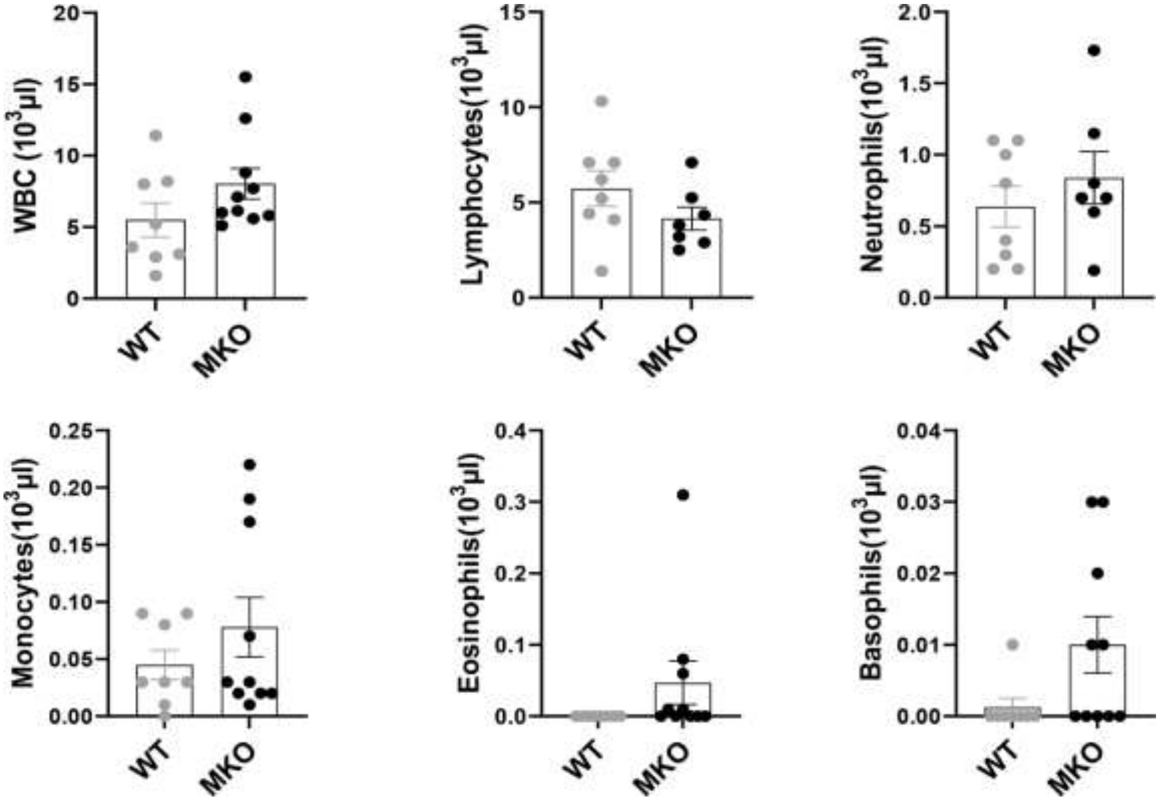

Figure S5. Circulatory leukocytes did not differ at baseline among WT and MKO mice.

Number of white blood cells (WBC), lymphocytes, neutrophils, monocytes, eosinophils and basophils in blood obtained from WT and MKO mice. *, $p<0.05$.

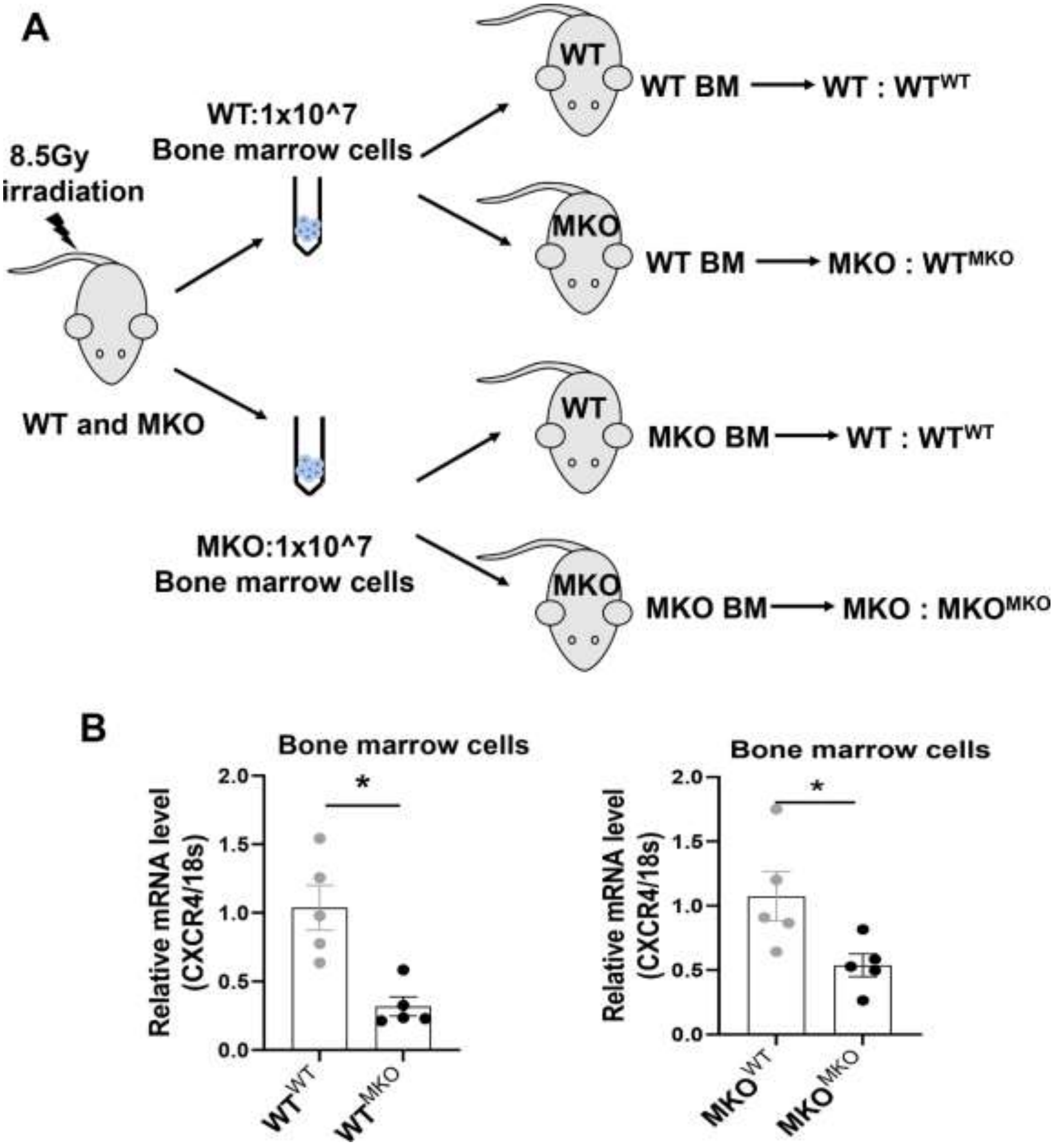

Figure S6. Bone marrow transplantation experiment.

(A) Schematic diagram of bone marrow transplantation experiment. **(B)** The mRNA levels of CXCR4 in the bone marrow cells of in WT^{WT}, WT^{MKO}, MKO^{WT}, and MKO^{MKO} mice. All data were analyzed using unpaired two-tailed student's t-test. *, $p<0.05$.

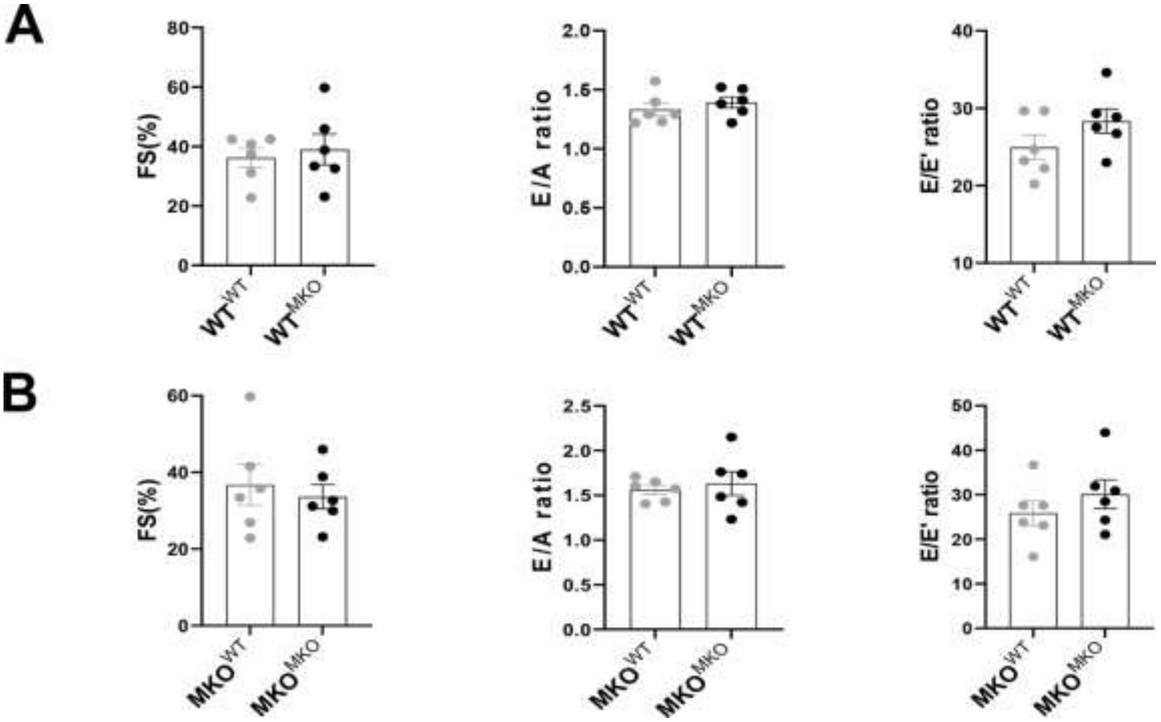

Figure S7. Baseline cardiac function showed no difference between WT and MKO mice after bone marrow transplantation.

(A) Measurement of FS%, E/A, and E/E’ in baseline level of WT^{WT} and WT^{MKO}. **(B)** Measurement of FS%, E/A, and E/E’ in baseline level of MKO^{WT} and MKO^{MKO}. WT^{WT}, n=6; WT^{MKO}, n=6; MKO^{WT}, n=6; MKO^{MKO}, n=6. Data were analyzed using unpaired two-tailed student’s t-test *, $p<0.05$.

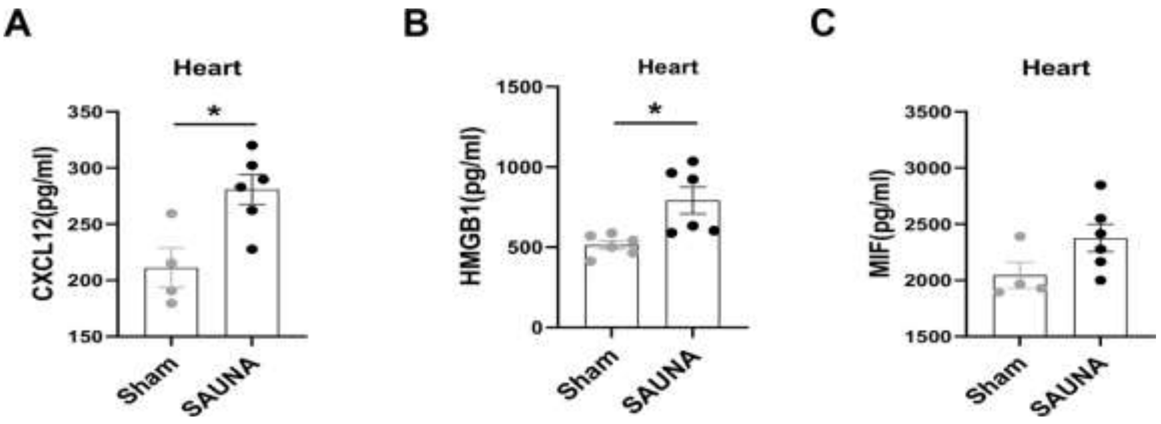

Figure S8. Ligands for CXCR4 were detected in the SAUNA-induced heart.

(A) Elisa analysis of the CXCL12 expression in the heart of sham and SAUNA group. Sham, n = 4; SAUNA, n = 6. **(B)** Elisa analysis of the HMGB1 expression in the heart of sham and SAUNA group. Sham, n = 4; SAUNA, n = 6. **(C)** Elisa analysis of the MIF expression in the heart of sham and SAUNA group. Sham, n = 4; SAUNA, n = 6.

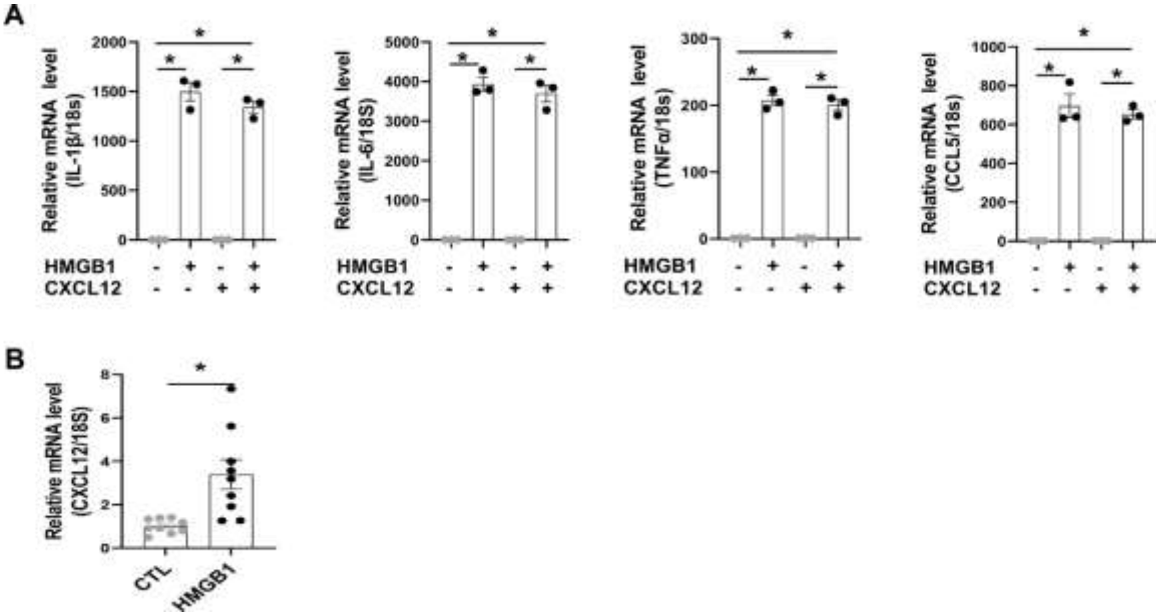

Figure S9. HMGB1 alone or in complex with CXCL12 triggered the production of pro-inflammatory cytokines in macrophages.

(A) The mRNA levels of IL-1β, IL-6, TNFα, and CCL5 in macrophages with or without CXCL12 after HMGB1 treatment. **(B)** The mRNA levels of CXCL12 in macrophages after HMGB1 treatment. Data were analyzed using unpaired two-tailed student's t-test *, $p<0.05$.

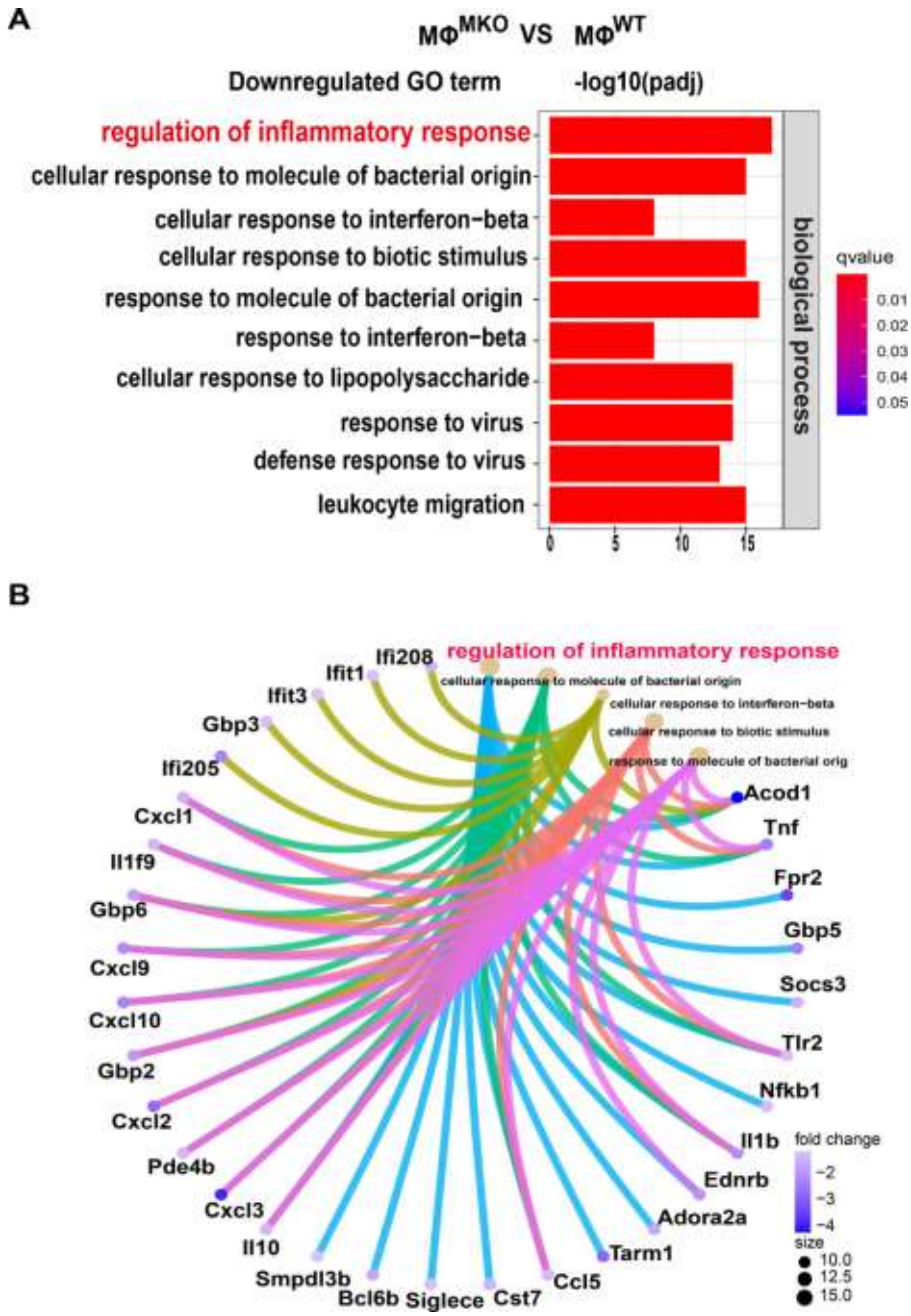

Figure S10. CXCR4 governs a pro-inflammatory phenotype in macrophages.

(A) GO enrichment analysis for biological process enriched in downregulated gene terms of MΦ^{WT} and MΦ^{MKO} after HMGB1 treatment. Top 10 of enriched GO term are listed. (B) Circos diagram of the indicated genes regulated in enriched biological processes in MΦ^{WT} and MΦ^{MKO} after HMGB1 treatment.

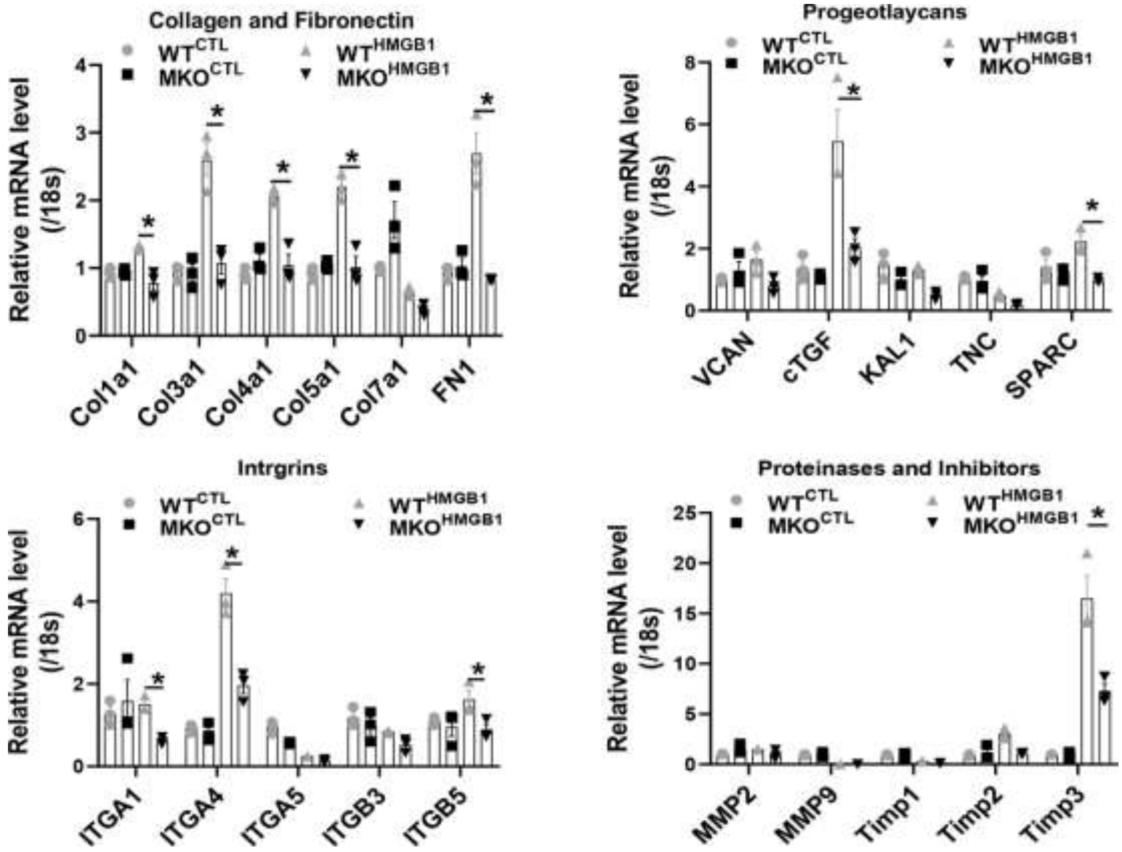

Figure S11. Deletion of CXCR4 downregulated ECM expression in macrophages

The mRNA levels of genes coding extracellular matrix proteins (collagen, fibronectin, proteoglycans, integrins, and matrix proteinases and inhibitors) in primary CFs co-cultured with $M\Phi^{WT}$ and $M\Phi^{MKO}$-CM with or without HMGB1 treatment. All data were analyzed using two-way ANOVA with Bonferroni's multiple comparisons test. *, $p<0.05$.

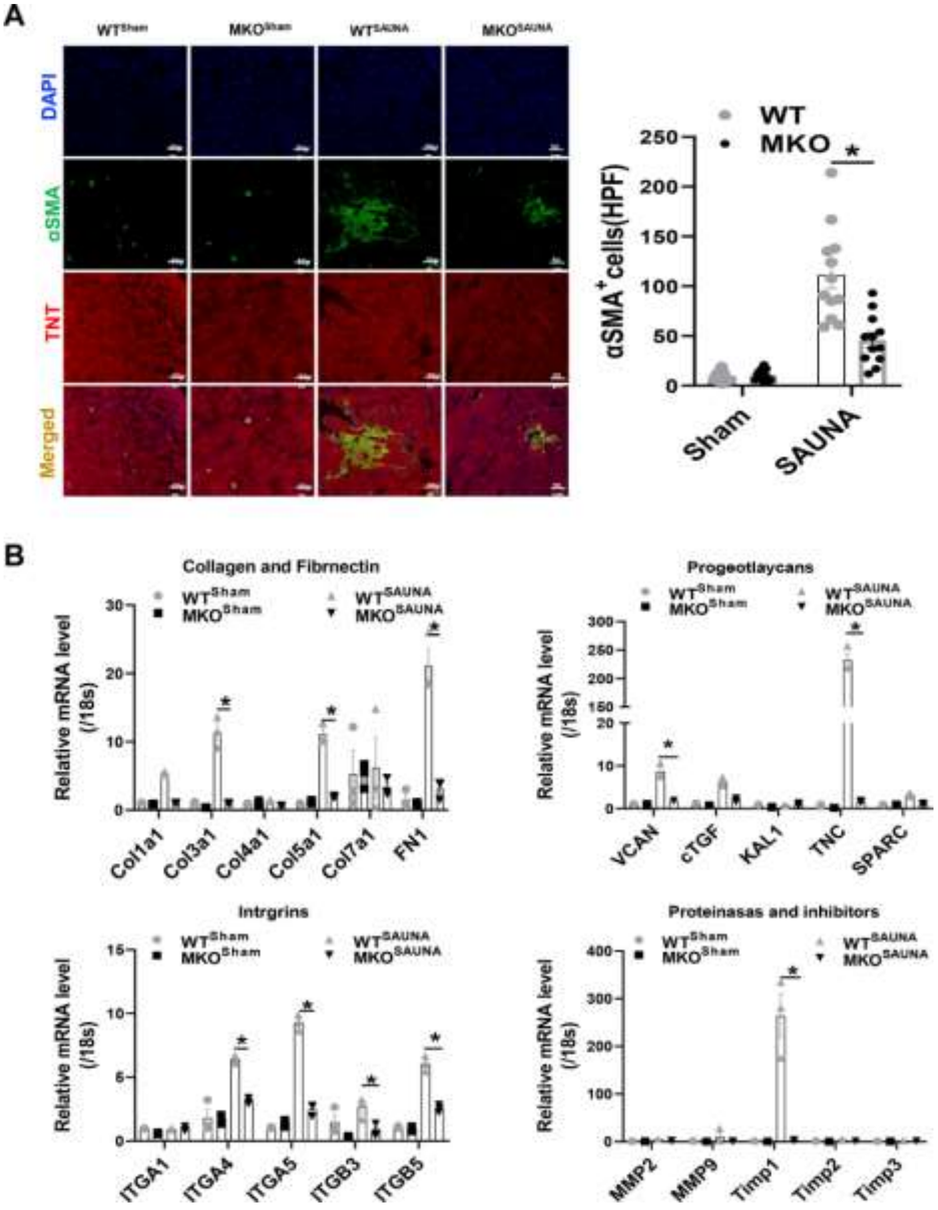

Figure S12. Deletion of CXCR4 impaired activation of cardiac fibroblasts and downregulated ECM expression in the SAUNA-induced heart..

(A) Immunofluorescence staining of anti-Troponin T (red) and anti-αSMA antibody (green) (DAPI, blue) in the heart of SAUNA induced WT and MKO mice. Scale bars=50 µm. **(B)**The mRNA levels of genes coding extracellular matrix proteins (collagen, fibronectin, proteoglycans, integrins, and matrix proteinases and inhibitors) in the heart of WT and MKO mice after sham or SAUNA operation.All data were analyzed using two-way ANOVA with Bonferroni's multiple comparisons test. *, $p<0.05$.

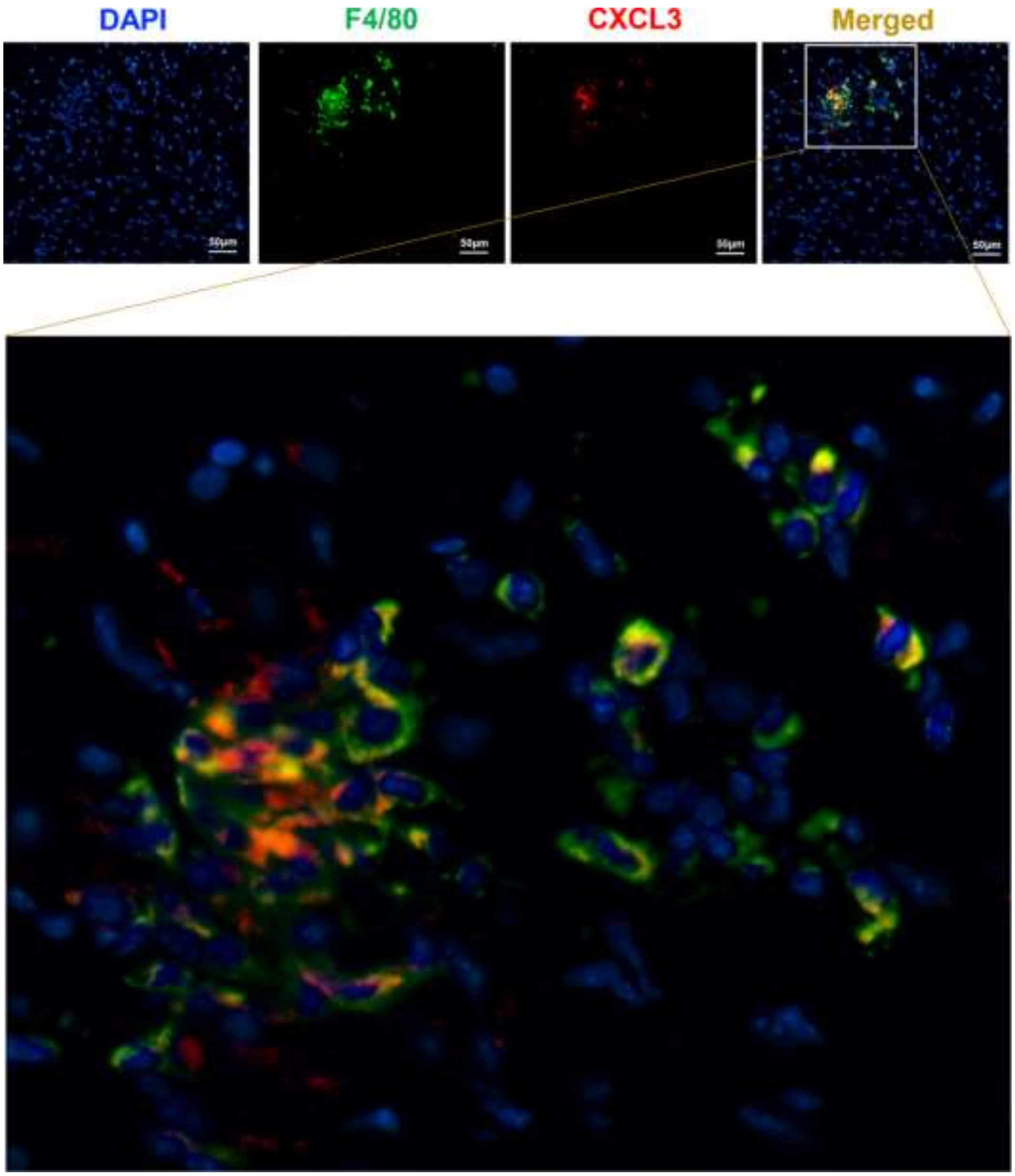

Figure S13. CXCL3 localized predominately with macrophages in SAUNA-exposed heart

Immunofluorescence staining of anti-CXCL3 (red) and anti-F4/80 antibody (green) (DAPI, blue) in SAUNA-exposed heart. Scale bars=50 μm.

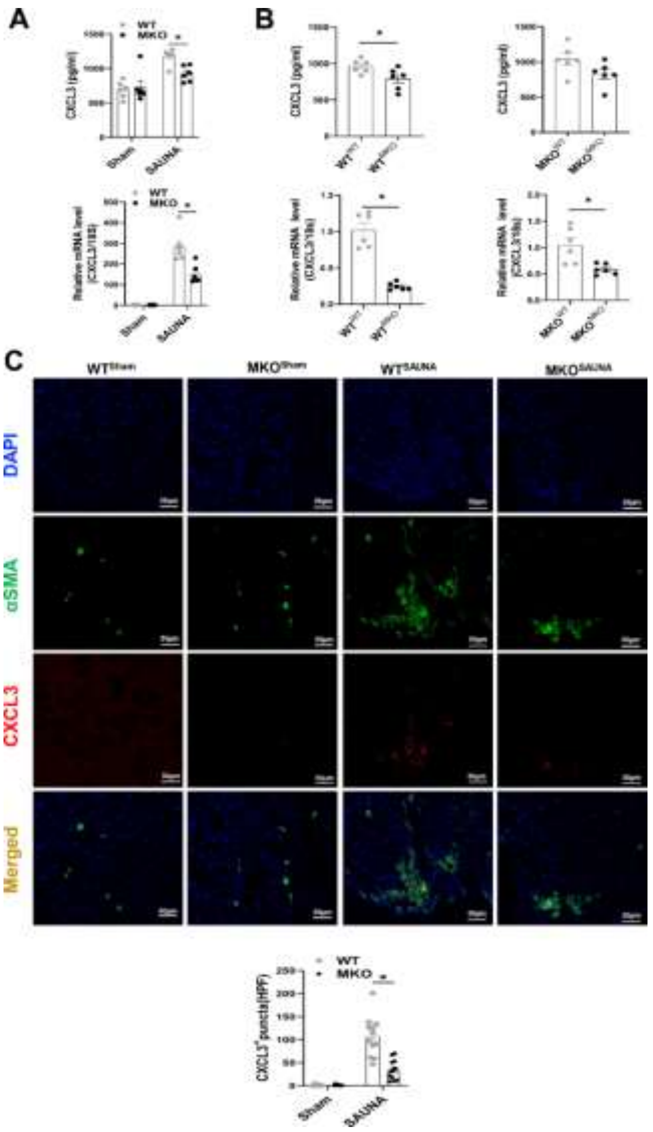

Figure S14. CXCR4 blockade macrophages suppressed CXCL3 in the SAUNA-induced heart. **(A)** Elisa analysis of the CXCL3 expression and mRNA levels of CXCL3 in the heart of WT and MKO mice after sham or SAUNA operation. **(B)** Elisa analysis of the CXCL3 expression and mRNA levels of CXCL3 in SAUNA-induced heart of WT and MKO mice after bone marrow transplantation. **(C)** Immunofluorescence staining of anti-CXCL3 (red) and anti-αSMA antibody (green) (DAPI, blue) in the heart of SAUNA induced WT and MKO mice. Scale bars=50 μm. WT^{sham}, n=6; MKO^{sham}, n=6; WT^{SAUNA}, n=6; and MKO^{SAUNA}, n=6. Data were analyzed using unpaired two-tailed student's t-test (**A and B)** and two-way ANOVA with Bonferroni's multiple comparisons test (**C**). *, $p<0.05$.

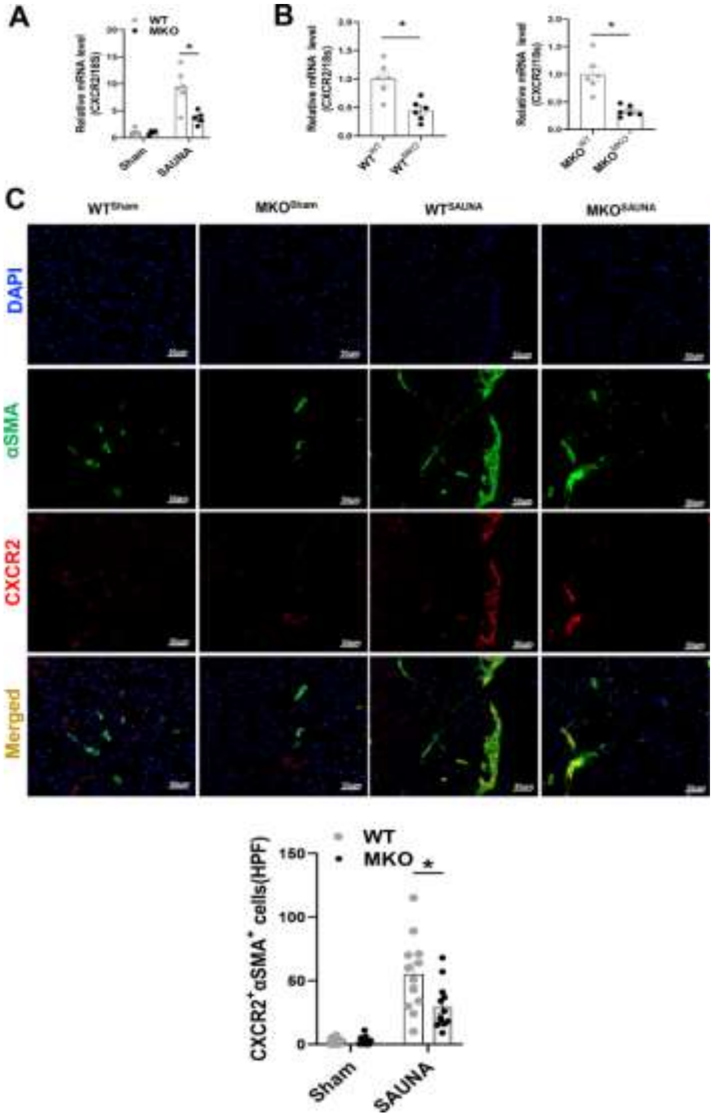

Figure S15. CXCR4 blockade macrophages suppressed CXCR2 in fibroblasts of SAUNA-induced heart.

(A) The mRNA level of the CXCR2 in the heart of WT and MKO mice after sham or SAUNA operation. **(B)** The mRNA level of the CXCR2 in SAUNA-induced heart of WT and MKO mice after bone marrow transplantation. **(C)** Immunofluorescence staining of anti-CXCR2 (red) and anti-αSMA antibody (green) (DAPI, blue) in the heart of SAUNA induced WT and MKO mice. Scale bars=50 µm. WT^{sham}, n=6; MKO^{sham}, n=6; WT^{SAUNA}, n=6; and MKO^{SAUNA}, n=6. Data were analyzed using unpaired two-tailed student's t-test (**A and B)** and two-way ANOVA with Bonferroni's multiple comparisons test (**C**). *, p<0.05.

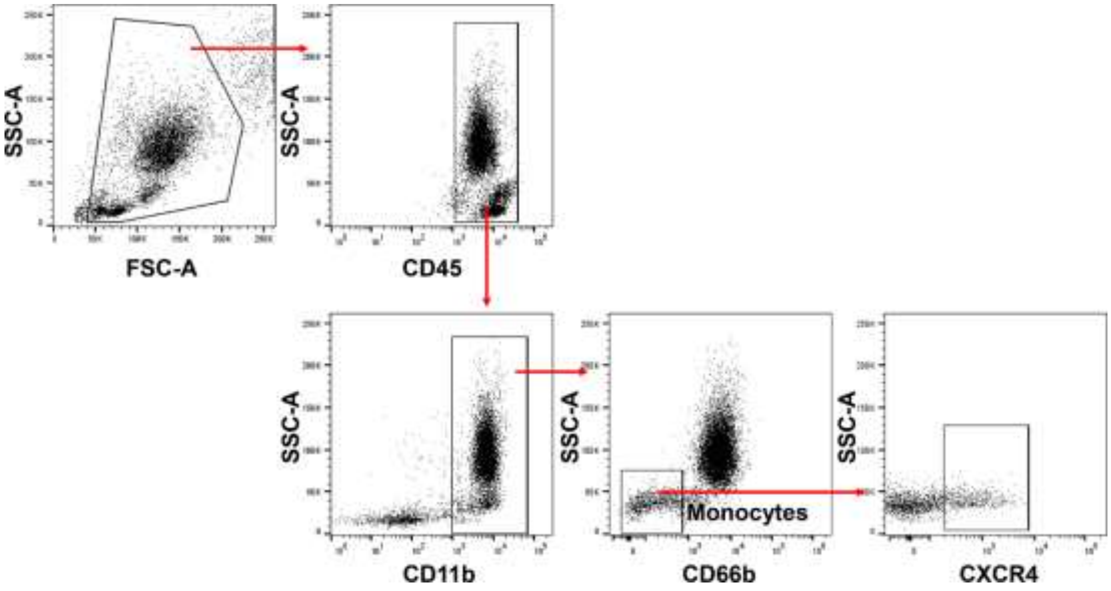

Figure S16. The immune cells in peripheral blood of HFpEF patients.

Gating strategy of CD45+ CD11b+ CD66B-CXCR4+ cells in peripheral blood of HFpEF patients.

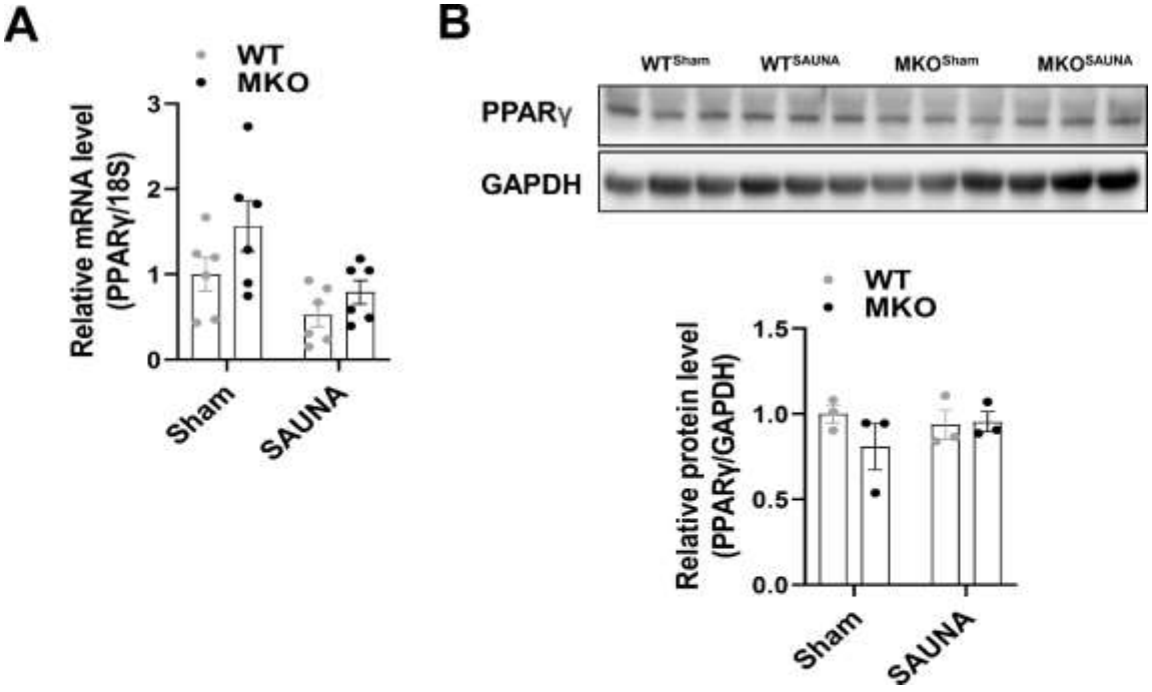

Figure S17. There was no significant difference in the PPARγ activity between SAUNA treated-WT or MKO hearts.

(A) The mRNA levels of PPARγ activity in SAUNA treated-WT or MKO hearts. (B) Western blot analysis of CXCR4 and PPARγ in SAUNA treated-WT or MKO hearts. Data were analyzed using unpaired two-tailed student's t-test (**A)** and two-way ANOVA with Bonferroni's multiple comparisons test (**B**).

Table S1. Name and sequence of primers sets for real-time RT-PCR

Gene name	Primer sequence
F4/80	Forward：GTGGAGGCAGTGATGCTCTT
	Reverse：TGGAAGCCCATAGCCAAAGG
CXCR4	Forward： TGCAGCAGGTAGCAGTGAAA
	Reverse：TGTATATACTCACACTGATCGGTTC
CXCL12	Forward：TGCATCAGTGACGGTAAACCA
	Reverse：TTCTTCAGCCGTGCAACAATC
18s	Forward：CCCAGTAAGTGCGGGTCATAA
	Reverse：CCGAGGGCCTCACTAAACC
IL-1β	Forward: AGGCTCATCTGGGATCCTCT
	Reverse: AGGCTCATCTGGGATCCTCT
IL-6	Forward: CCACTTCACAAGTCGGAGGCTTA
	Reverse: GCAAGTGCATCATCGTTGTTCATAC
TNFα	Forward: ACAAGATGCTGGGACAGTGA
	Reverse: ACCTGACCACTCTCCCTTTG
CCL5	Forward：GCTGCTTTGCCTACCTCTCC
	Reverse：TCGAGTGACAAACACGACTGC
Col1a1	Forward：GCTCCTCTTAGGGGCCACT
	Reverse：CCACGTCTCACCATTGGGG
Col3a1	Forward：CTGTAACATGGAAACTGGGGAAA
	Reverse：CCATAGCTGAACTGAAAACCACC
Col4a1	Forward：TCCGGGAGAGATTGGTTTCC
	Reverse：CTGGCCTATAAGCCCTGGT
Col5a1	Forward：CTTCGCCGCTACTCCTGTTC

	Reverse：CCCTGAGGGCAAATTGTGAAAA
Col7a1	Forward：GCCCAGAGATAGAGTGACCTG
	Reverse：CGCACTTCTCGAAAGTTGCTG
FN1	Forward：ATGTGGACCCCTCCTGATAGT
	Reverse：GCCCAGTGATTTCAGCAAAGG
VCAN	Forward：TTTTACCCGAGTTACCAGACTCA
	Reverse：GGAGTAGTTGTTACATCCGTTGC
cTGF	Forward：CGCTGTGATGACGGTGGTTT
	Reverse：CCTGGCACCTGTATTCTCCTG
KAL1	Forward：CAGCTAATGAATGGCGTTCTAGG
	Reverse：CTTAGGTTGATAACGAGGGCAG
TNC	Forward：ACGGCTACCACAGAAGCTG
	Reverse：ATGGCTGTTGTTGCTATGGCA
SPARC	Forward：GTGGAAATGGGAGAATTTGAGGA
	Reverse：CTCACACACCTTGCCATGTTT
ITGA1	Forward：CCTTCCCTCGGATGTGAGTCA
	Reverse：AAGTTCTCCCCGTATGGTAAGA
ITGA4	Forward：GATGCTGTTGTTGTACTTCGGG
	Reverse：ACCACTGAGGCATTAGAGAGC
ITGA5	Forward：CTTCTCCGTGGAGTTTTACCG
	Reverse：GCTGTCAAATTGAATGGTGGTG
ITGB3	Forward：CCACACGAGGCGTGAACTC
	Reverse：CTTCAGGTTACATCGGGGTGA
ITGB5	Forward：GCTGCTGTCTGCAAGGAGAA

	Reverse：AAGCAAGGCAAGCGATGGA
TIMP1	Forward：GCAACTCGGACCTGGTCATAA
	Reverse：CGGCCCGTGATGAGAAACT
TIMP2	Forward：TCAGAGCCAAAGCAGTGAGC
	Reverse：GCCGTGTAGATAAACTCGATGTC
TIMP3	Forward：CTTCTGCAACTCCGACATCGT
	Reverse：GGGGCATCTTACTGAAGCCTC
ACTA2	Forward：GTCCCAGACATCAGGGAGTAA
	Reverse：TCGGATACTTCAGCGTCAGGA
POSTIN	Forward：CCTGCCCTTATATGCTCTGCT
	Reverse：AAACATGGTCAATAGGCATCACT
DDR	Forward：GCTCTCCAATCCGGCCTAC
	Reverse：CGGGCTCCATATAGTCCCCA
PDGFRA	Forward：TCCATGCTAGACTCAGAAGTCA
	Reverse：TCCCGGTGGACACAATTTTTC
TCF21	Forward：CCCACTAAGAAAAGCCCGCTC
	Reverse：CCGTTCTCGTACTTGTCGTTG
CXCL3	Forward：CAGTGCCTGAACACCCTACC
	Reverse：GGACTTGCCGCTCTTCAGTA
BNP	Forward：GGTGCTGTCCCAGATGATT
	Reverse：GCCATTTCCTCCGACTTT
ANP	Forward: GCCATTTCCTCCGACTTT
	Reverse：TCCAGGTGGTCTAGCAGGTT
PPARγ	Forward: GGAAGACCACTCGCATTCCTT

	Reverse：GTAATCAGCAACCATTGGGTCA
PPARα	Forward: AGAGCCCCATCTGTCCTCTC
	Reverse：ACTGGTAGTCTGCAAAACCAAA
PPARβ	Forward: TCCATCGTCAACAAAGACGGG
	Reverse：ACTTGGGCTCAATGATGTCAC
Lysm-Cre	oIMR3066: CCC AGA AAT GCC AGA TTA CG
	oIMR3067：CTT GGG CTG CCA GAA TTT CTC
	oIMR3068：TTA CAG TCG GCC AGG CTG AC
CXCR4-lxop	10378: CCA CCC AGG ACA GTG TGA CTC TAA
	10379：GAT GGG ATT TCT GTA TGA GGA TTA GC

Table S2. RNA-seq result of the top 18 significant genes in $M\Phi^{WT}$ and $M\Phi^{MKO}$ under HMGB1 stimulation.

Gene_id	Gene_name	MKO	WT	log2Fold Change	padj
ENSMUSG00000095478	Gm9824	180.5134	351.3764	-0.96047	0.002101
ENSMUSG00000061983	Rps12	3255.999	4651.913	-0.51488	0.002747
ENSMUSG00000029379	Cxcl3	2.424679	36.65793	-3.9104	0.032014
ENSMUSG00000021298	Gpr132	232.3973	408.1813	-0.81156	0.0391
ENSMUSG00000062169	Cnih4	1103.998	1406.751	-0.34956	0.127725
ENSMUSG00000014444	Piezo1	8705.321	7426.549	0.229215	0.199229
ENSMUSG00000021453	Gadd45g	2447.341	1923.054	0.347637	0.25559
ENSMUSG00000050071	Bex1	1546.675	1080.197	0.517379	0.25559
ENSMUSG00000041453	Rpl21	864.0283	1830.075	-1.08283	0.25559
ENSMUSG00000040253	Gbp7	237.3416	414.9638	-0.80507	0.25559
ENSMUSG00000004891	Nes	1078.15	797.9487	0.43364	0.25559
ENSMUSG00000027523	Gnas	917.1409	686.7162	0.417587	0.25559
ENSMUSG00000032915	Adgre4	123.6392	241.3621	-0.9637	0.25559
ENSMUSG00000029066	Mrpl20	743.8891	948.4467	-0.35012	0.25559
ENSMUSG00000041992	Rapgef5	174.4833	285.0491	-0.7075	0.25559
ENSMUSG00000022048	Dpysl2	482.6345	681.1266	-0.49636	0.25559
ENSMUSG00000031604	Msmo1	1726.224	2296.406	-0.41153	0.284661
ENSMUSG00000023367	Tmem176a	485.2247	687.2435	-0.50149	0.284661
ENSMUSG00000025934	Gsta3	46.67984	116.6869	-1.31995	0.284661
ENSMUSG00000068396	Rpl34-ps1	286.3335	424.6136	-0.56772	0.284661

Table S3. Patient clinical baseline characteristics

	normal individuals (n=13)	HFpEF patients (n=23)	P valve (*, p<0.05)
**Age (year)**	32.31±11.96	59.78±9.46	<0.0001*
**Gender（male/female）**	6：7	15：8	0.3101
**Comorbidity**			
Hypertension (n, %)	0 (0%)	23，100%	<0.0001*
Diabetes (n, %)	0 (0%)	0 (0%)	
Coronary heart disease (n, %)	0 (0%)	0 (0%)	
Peripheral arterial disease (n, %)	0 (0%)	0 (0%)	
**Doppler echocardiography**			
LVID. d (mm)	4.40±0.32	4.81±0.64	0.0444*
LA (mm)	3.00±0.30	3.62±0.48	0.0002*
LVEF (%)	65.41±4.31	62.35±9.78	0.3057
E/A	1.60±0.35	0.76±0.15	<0.0001*
E/E’	7.01±1.82	12.32±3.73	<0.0001*
**Pro-BNP (pg/ml)**	29.86±12.68	1435.76±1650.47	0.0421*
**Blood routine**			
Neutrophil absolute value(10^9/L)	3.50±1.04	3.84±1.31	0.2911
Lymphocyte absolute value(10^9/L)	1.56±0.63	1.96±1.17	0.2129
Monocyte absolute value(10^9/L)	0.39±0.10	0.58±0.23	0.0093*

Table S4. The antibodies and cytokines used in the study

Name Company Item	Name Company Item No
For western blot:	
GAPDH	1:3000, Cell Signaling Technology, Danvers, MA, USA, 5174
aSMA	1:1000, Cell Signaling Technology, Danvers, MA, USA, 48938
Fibronectin	1:1000, Abcam, Cambridge, MA, USA, ab2413
PPARγ	1:1000, Cell Signaling Technology, Danvers, MA, USA, 2435
CXCR2	1:1000, Abcam, Cambridge, MA, USA, ab65968
NF-κB p65	1:1000, 8242, Cell Signaling Technology, Danvers, MA, USA
pNF-κB p65	1:1000, 3033, Cell Signaling Technology, Danvers, MA, USA
MEK	1:1000, ET1602-3, Huabio, China
pMEK	1:1000, ET1612-40, Huabio, China
Erk	1:1000, RT1484, Huabio, China
pErk	1:1000, ET1603-22, Huabio, China
For immunofluorescence:	
F4/80	1:300, Abcam, Cambridge, MA, USA, 6640
aSMA	1:300, Cell Signaling Technology, Danvers, MA, USA, 48938
CXCL3	1:1000, Abcam, Cambridge, MA, USA, ab220431
CXCR4	1:1000, Abcam, Cambridge, MA, USA, ab181020
For flow cytometry	
CD45	1:1000, BD Biosciences, San Jose, CA, USA, 557659, 555482
CD11b	1:1000, BD Biosciences, San Jose, CA, USA, 553312, 557657
CD66B	1:1000, BioLegend, San Diego, CA, USA, 305118
F4/80	1:1000, BD Biosciences, San Jose, CA, USA,123108, 123114
Ly6G	1:1000, BD Biosciences, San Jose, CA, USA,560602
CXCR4	1:1000, BD Biosciences, San Jose, CA, USA,551510, 551966
CCR2	1:1000, BD Biosciences, San Jose, CA, USA, 150608
Others	

Aldosterone	0.3ug/h, Sigma-Aldrich Co., St. Louis, Missouri, 706035
Recombinant Mouse CXCL3	10ng/ml, MCE, Shanghai, China, HY-P7153
Recombinant Mouse HMGB1	1ug/ml, Abcam, Cambridge, MA, USA, ab181949
Recombinant Mouse M-CSF	10ng/ml, PEROTECH, New Jersey, USA, 315-02
Anti-CXCL3 neutralizing antibody	0.5ug/ml, R&D, Minneapolis, MN, USA, AF5568
GW9662	10nM, MCE, Shanghai, China, HY-16578
GW1929	10nM, MCE, Shanghai, China, HY-15655
SB225002	10nM, MCE, Shanghai, China, HY-16711
PD98059	10nM, MCE, Shanghai, China, HY-12028
